# Post-hoc standardisation of parametric T1 maps in cardiovascular magnetic resonance imaging: a proof-of-concept

**DOI:** 10.1016/j.ebiom.2024.105055

**Published:** 2024-03-14

**Authors:** Darian Viezzer, Thomas Hadler, Jan Gröschel, Clemens Ammann, Edyta Blaszczyk, Christoph Kolbitsch, Simone Hufnagel, Riccardo Kranzusch-Groß, Steffen Lange, Jeanette Schulz-Menger

**Affiliations:** a Charité – Universitätsmedizin Berlin, Corporate Member of Freie Universität Berlin and Humboldt-Universität zu Berlin, ECRC Experimental and Clinical Research Center, Lindenberger Weg 80, 13125 Berlin, Germany; b Working Group on Cardiovascular Magnetic Resonance, Experimental and Clinical Research Center, A Joint Cooperation Between the Charité – Universitätsmedizin Berlin and the Max-Delbrück-Center for Molecular Medicine, Berlin, Germany; c DZHK (German Centre for Cardiovascular Research), Partner Site Berlin, Berlin, Germany; d Physikalisch-Technische Bundesanstalt (PTB), Braunschweig and Berlin, Germany; e Universitätsklinikum Schleswig-Holstein, Klinik für Radiologie und Nuklearmedizin, Lübeck, Germany; f Hochschule Darmstadt (University of Applied Sciences), Faculty for Computer Sciences, Darmstadt, Germany; g Helios Hospital Berlin-Buch, Department of Cardiology and Nephrology, Berlin, Germany

**Keywords:** Cardiovascular Magnetic Resonance, T1 Mapping, Standardisation, Healthy Volunteers, Left Ventricular Hypertrophy, Amyloidosis

## Abstract

**Background:**

In cardiovascular magnetic resonance imaging parametric T1 mapping lacks universally valid reference values. This limits its extensive use in the clinical routine. The aim of this work was the introduction of our self-developed Magnetic Resonance Imaging Software for Standardization (MARISSA) as a post-hoc standardisation approach.

**Methods:**

Our standardisation approach minimises the bias of confounding parameters (CPs) on the base of regression models. 214 healthy subjects with 814 parametric T1 maps were used for training those models on the CPs: age, gender, scanner and sequence. The training dataset included both sex, eleven different scanners and eight different sequences. The regression model type and four other adjustable standardisation parameters were optimised among 240 tested settings to achieve the lowest coefficient of variation, as measure for the inter-subject variability, in the mean T1 value across the healthy test datasets (HTE, N = 40, 156 T1 maps). The HTE were then compared to 135 patients with left ventricular hypertrophy including hypertrophic cardiomyopathy (HCM, N = 112, 121 T1 maps) and amyloidosis (AMY, N = 24, 24 T1 maps) after applying the best performing standardisation pipeline (BPSP) to evaluate the diagnostic accuracy.

**Findings:**

The BPSP reduced the COV of the HTE from 12.47% to 5.81%. Sensitivity and specificity reached 95.83% / 91.67% between HTE and AMY, 71.90% / 72.44% between HTE and HCM, and 87.50% / 98.35% between HCM and AMY.

**Interpretation:**

Regarding the BPSP, MARISSA enabled the comparability of T1 maps independently of CPs while keeping the discrimination of healthy and patient groups as found in literature.

**Funding:**

This study was supported by the 10.13039/501100002347BMBF / 10.13039/100010447DZHK.

**Research in context**

**Evidence before this study**

Although parametric T1 mapping is considered as an important method in cardiovascular magnetic resonance (CMR), the lack of universal valid reference values has been mentioned in many studies as an obstacle to fully utilise T1 mapping in different cohorts, multi-side studies or current guidelines. Hence, literature research covering the time frame from September 2016 until October 2023 in PubMed and Google Scholar for articles on T1 mapping in CMR revealed only the z-Score approach as a way to define comparable values. However, the necessity of a healthy cohort examination whenever a technical change is performed, high volatility in the standard deviation of healthy volunteer examination and lack of accessibility seems to be obstacles of this approach.

**Added value of this study**

Our work introduces the Magnetic Resonance Imaging Software for Standardization (MARISSA) as an approach for the post-hoc standardisation of parametric T1 maps in CMR. As this standardisation pipeline can capture different settings, we analysed 240 different settings in a two-step approach and evaluated the best performing one. We were able to show that the choice of the pipeline setting is crucial for the success of the standardisation and that this proof-of-concept including the parameters age, sex, scanner and sequence is already good enough to differentiate healthy volunteers from patients with hypertrophic cardiomyopathy and amyloidosis as in a highly controlled intra-scanner-intra-sequence setting.

**Implications of all the available evidence**

Our results demonstrate that a post-hoc standardisation of parametric T1 maps is feasible. The implementation as a python software with a graphical user interface makes the standardisation procedure directly available and shareable on any common operating system. Although the described approach with four considered confounding parameters already allowed a comparable discrimination of two cardiovascular diseases from a healthy cohort, this work is a proof-of-concept that needs further investigations on more scanners, sequences and diseases but also other confounding parameters. Compared to the z-score, our standardisation pipeline does not require a re-examination of a healthy reference cohort on each site whenever a technical change occurs. This reduces the effort and costs to increasingly enable parametric T1 mapping. Consequently, this work is a further step forward to strengthen the establishment of parametric T1 mapping in the clinical routine, which in turn helps to improve the detection of cardiovascular diseases.

## Introduction

Cardiovascular magnetic resonance (CMR) is recommended as the non-invasive imaging modality of choice for myocardial tissue characterisation in cardiovascular diseases (CVD).^1^, ^2^, ^3^ This characterisation is enabled by quantitative methods such as parametric T1 mapping.^4^^,^^5^ Its integration into clinical CMR routine protocol recently showed improved diagnostic accuracy for the detection of CVDs.^6^ Although parametric T1 mapping is already increasingly used in clinical routine and turned from a research to a product sequence, reproducibility is limited to intra-institutional reference values.^4^^,^^7^ This lack of universally applicable reference values is a major obstacle for a stronger assertiveness of parametric T1 mapping in the clinical routine and is caused by subject specific, technological and post-processing procedure variations.^7^, ^8^, ^9^ These variations act as confounding parameters (CPs) on parametric T1 maps and thereby on the quantitative outcome, which in turn potentially influence the treatment of CVDs. Hence, every change in hard- or software may require new local reference values and thus a re-examination of a healthy reference cohort.

Recently, the reproducibility of parametric T1 mapping was validated across different scanners if CPs such as manufacturer, field strength, acquisition schemes and post-processing were kept constant.^10^ However, the technical setups across institutions are manifold and cannot be globally aligned by force. As the influence of CPs such as age and sex,^11^^,^^12^ sequence variants^13^^,^^14^ or scanner models^12^ on parametric T1 mapping were described in the literature, universal valid reference values are required to consider and consequently to minimise the induced CP's bias.

All in all, there is a gap of defining universal valid reference values in parametric T1 mapping based on a generalised approach. For that reason, the aim of this work is to introduce a generic post-hoc standardisation pipeline that enables comparability while maintaining diagnostic accuracy and reducing the amount of necessary healthy volunteer examinations. We propose that standardisation is enabled by estimating the impact of a CP relatively to a reference CP value. Consequently, parametric T1 mapping values are post-hoc transformable into values of a reference CP environment. This proposed transformation is embedded in the self-developed open-source Magnetic Resonance Imaging Software for Standardization (MARISSA) that is made available with this work.

## Methods

In this work the four CPs: age,^11^^,^^12^ sex,^11^^,^^12^ scanner^12^ and sequence variant^13^^,^^14^ were chosen from literature to show a proof-of-concept for the proposed post-hoc standardisation of parametric T1 maps. This includes the introduction of MARISSA as a software tool to setup those post-hoc standardisation pipelines and to demonstrate the diagnostic quality after standardisation. Therefore, the used data collection included three cohorts: healthy volunteers (Healthy), patients with left ventricular hypertrophy (LVH) including hypertrophic cardiomyopathy (HCM) and patients with amyloidosis (AMY). Both patient cohorts were included on account of a statistical significant differentiation from healthy volunteers in native T1 mapping with considerably higher T1 values in AMY and on average higher but partly overlapping ranges in HCM.^15^ The following first sub-section Dataset describes the used data collection of the three included cohorts. A part of Healthy (Healthy train datasets/HTR) was used in a first step to estimate the individual CP impact. As different strategies exist to estimate the CP induced bias, the Confounding Parameters Impact Estimation (CPIE) sub-section covers detailed information about the different estimation strategies. The following sub-section on the Best Performing Standardisation Pipeline (BPSP), describes the evaluation of the best performing CPIE among all tested strategies with respect to the remaining healthy volunteers (Healthy test datasets/HTE). The before last sub-section on Diagnostic Implication (DI) covers the evaluation of the diagnostic accuracy and intra-subject differences after applying the BPSP on the said HTE as well as the HCM and AMY cohorts and includes the Statistics. Finally, the Implementation sub-section describes briefly the MARISSA structure followed by the Ethical approval and Role of the funding source.

### Dataset

**Fig. 1:**
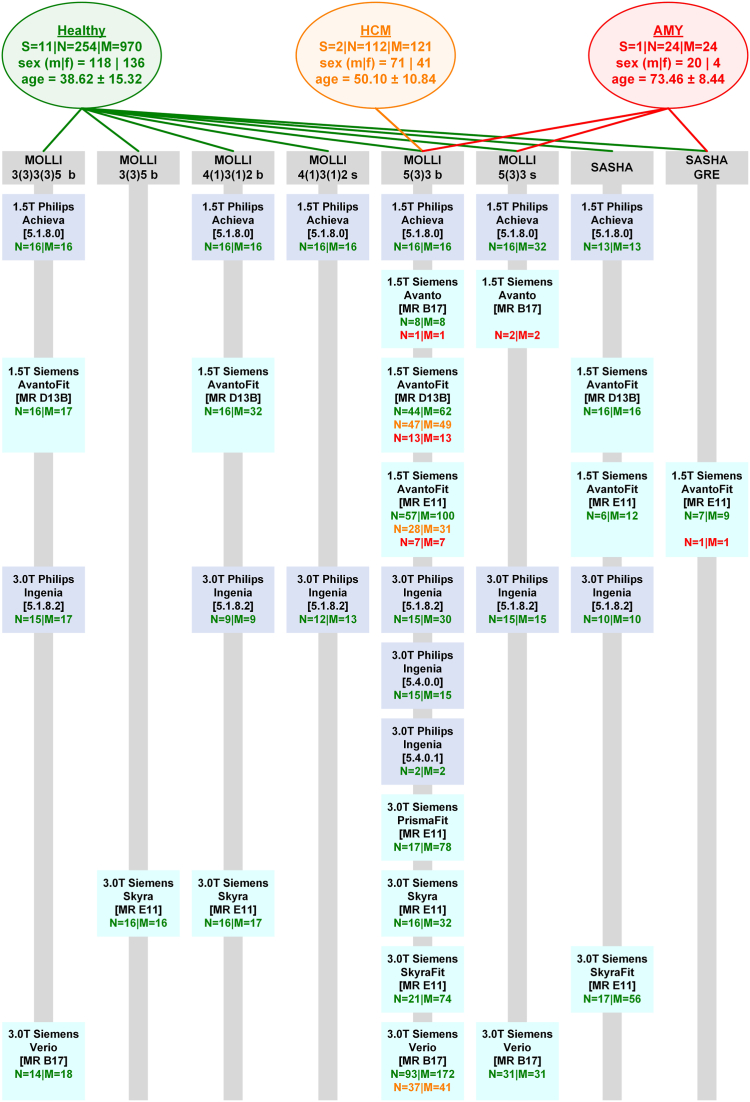
Dataset Overview – The general overview of the Healthy (green), patients with left ventricular hypertrophy including hypertrophic cardiomyopathy (HCM, orange) and patients with amyloidosis (AMY, red) cohorts are given with S: number of studies, N: number of subjects, M: number of midventricular native parametric T1 maps, m: males and f: females. The age is given as mean ± standard deviation. The grey bars represent respective parametric T1 mapping sequences and the turquoise and blue boxes represent a scanner. For each scanner-sequence combination the number of subjects and respective T1 maps is itemised.

The included retrospective data collection of the three cohort groups: Healthy, HCM and AMY consisted of midventricular slices only and originated from previous and ongoing studies of our working group or in which our working group participated until June 2023 considering scanners that are part of the Berlin CMR research network.^10^ Age was the only numerical CP; all other considered CPs were categorical. The various origins in the data collection enabled variability in the concerned CPs while some individuals received multiple measurements, i.e. different sequences and/or scanners. Fig. 1 shows an overview of the total numbers in each cohort as well as the variation in the four concerned CPs. A detailed breakdown of the underlying data for each scanner-sequence combination for both sexes is provided in the Supplemental Material S1.

The segmentation of all parametric T1 maps was performed automatically with a subsequent visual inspection by two experts (JG and EB). First, all T1 maps were segmented using the cropU cascaded model^16^ and a research deep learning segmentation model provided by Siemens Healthcare (version 21 hotfix, Siemens Healthcare GmbH, Erlangen, Germany). The expert chose the best segmentation out of either model or the intersection of both. If none was considered accurate enough by the experts, the segmentation was performed manually in cvi42 (version 5.13.7, Circle Cardiovascular Imaging, Calgary, Canada) as previously reported.^10^

### Confounding Parameters Impact Estimation (CPIE)

The CPIE is a regression model training based on the HTR and represents the central part of the proposed standardisation pipeline. For that reason, the Healthy data collection were split into 85% training and 15% testing with respect to the number of subjects per study. Thus the HTR consisted of 214 subjects (814 T1 maps, 100 males / 114 females and 38.46 ± 15.20 years) while the HTE consisted of 40 subjects (156 T1 maps, 18 males / 22 females, 39.50 ± 15.89 years).

The CP impact can be solely estimated relatively to a reference as the absolute true T1 mapping value is unknown due to an intrinsic lack of accuracy or precision in T1 acquisition methods.^9^ Therefore, we propose to define for each CP a reference value that is assumed with no bias. In this work, the reference CP values were set to 18 years, male, 3.0T Siemens Verio [syngo MR B17] and T1 Map MOLLI 5(3)3 b for the concerned CPs age, sex, scanner and sequence variant respectively.

Consequently, the regression estimates the difference between the examined apparent T1 mapping value in the according CP environment and the target T1 mapping value in the reference CP environment. The target T1 mapping value is defined as the mean T1 value of HTR subjects whose concerned CP value matches the reference CP value. During the fitting of the regression the difference between apparent and target T1 mapping value represents the dependent and the CP value(s) represent the independent variable(s). Whether one or multiple CPs are taken into account depends on the strategy setting. Table 1 lists an overview of possible settings for the standardisation pipeline that are explained in the following.

**Table 1:** Standardisation pipeline setting – Each pipeline setting can capture exact one of the given possible values.

Setting	Options
regression-type	extra-trees (ETR) linear linear support vector (LSVR) random-forest (RFR)
y-type	absolute relative
mode	cascaded ensemble individual
bins	1 to minimum number of segmented pixels
cluster-type	agglomerative average agglomerative complete agglomerative single agglomerative ward equal distant equal size gaussian mixture k-means

Each CPIE model is described by the regression-type, y-type, mode, bins and cluster-type. The linear regression is the most basic regression model used in a variety of disciplines^17^ while the linear support vector regression^18^ (LSVR) was implemented as an alternative linear model. Additionally, the random-forest^19^ regression (RFR) and extra-trees^20^ regression (ETR) were implemented as those are assumed to handle non-linear relationships better than linear models. All regression models were based on the scikit-learn package^21^ and setup in the default setting, except for RFR and ETR, where the number of estimator trees were increased to 1000. While an increased number of trees enables an improved performance, an overfitting is excluded by its intrinsic structure.^19^

The chosen regression-type can either estimate the difference between apparent and target T1 value in absolute (ms) or relative (%) values according to the y-type setting. While the absolute case shifts all values equally such that the intrinsic value spread maintains, the relative case weights the shift according to the absolute T1 value.

The mode, finally, defines whether a regression is performed for each CP individually (individual and cascaded) or all at once (ensemble). The individual mode fits a regression for each CP individually and independent. Any other CPs are kept constant to minimise their impact on the regression. Consequently, only a small portion of the HTR can be used for each CP regression fit. The cascaded mode works the same for the first CP regression but iteratively standardise the HTR data, such that subsequent CPs must not consider previously fitted CPs. Therefore, the amount of useable data increases for later CPs. It is important to note that the outcome depends on the order of the considered CPs. Finally, the ensemble mode takes all CPs at once. The ensemble mode is expected to handle cross-dependent CPs best. Categorical variables are converted into category numbers in the ensemble mode, whereas in individual and cascaded mode each category receives its own regression model.

While regression-type, y-type and mode directly affects the regression model, the setting of a bin larger than one accounts for cross-dependencies between the CP value and the apparent T1 value. It is currently unknown, if higher T1 values are differently affected by a CP than lower ones. The setting of a bin larger than one requires the choice of a clustering algorithm that clusters the T1 values into bins. The different agglomerative clustering algorithms, the Gaussian mixture and k-means clustering were taken from the scikit-learn package^21^ while equal distant and equal size clustering were self-implemented. For equal distant clustering all bins have the same width while for equal size clustering the T1 values are sorted and the same number of T1 values are used for each bin. Although the number of bins is mainly limited by the smallest possible number of segmented pixels, which would represent each pixel as its own cluster, we recommend ten bins or fewer as otherwise the number of T1 values in each bin is too small to be representative.

In the light of the standardisation pipeline, CPIE outputs the estimated bias and is therefore an integrative part of the pipeline. First, a T1 mapping dataset in Digital Imaging and Communications in Medicine (DICOM) format with a corresponding segmentation mask is expected as input. After extracting the segmented apparent T1 values, the CPIE is applied to estimate the CP induced bias. This bias is then subtracted from the apparent T1 values and the resulting standardised T1 values are returned as output.

### Best performing standardisation pipeline (BPSP)

As the optimal standardisation pipeline setting is unknown, CPIE were evaluated for 240 different settings in two steps. First, bins were set to one, hence, cluster-type had no impact and all 24 combinations of regression-type, y-type and mode were fitted. The resulting standardisation pipelines were evaluated with respect to the coefficient of variation (COV, Equation (1)) of the mean T1 time in the HTE.

(Equation 1) $$COV=\frac{\sigma }{\mu }$$

The lower the COV, the less variability across the subjects of the HTE exist and, consequently, a better pipeline performance can be assumed. Considering the top three performing pipelines of this first step, fitting was performed for all combinations of two to ten bins and clustering algorithms resulting in additional 216 pipelines. The BPSP was evaluated as the one out of the 240 fitted standardisation pipelines with the lowest COV in the HTE group.

### Diagnostic Implication (DI)

While the evaluation of the BPSP accounted for a minimisation of the inter-healthy-subject variability, the DI step assessed the discriminability between the healthy test cohort and patients. Therefore, the HTE, HCM and AMY cohort were standardised with the BPSP. A progression plot was used to show the value progression from before to after standardisation.

### Statistics

All available retrospective datasets were included that were diagnosed as either Healthy, HCM or AMY. Age and sex were self-reported and all subjects were 18 years or older. Non-midventricular and contrast enhanced parametric T1 maps were excluded. The data were randomized, blinded and checked for artifacts by two experts (JG and EB).

The outcome statistics of the post-hoc standardisation is integrated in the DI. This includes the boxplots for each cohort before and after standardisation indicating the respective value spread. Further, confidence intervals (CIs) were calculated and statistics between the cohorts after standardisation were tested with an independent t-test and ANOVA test if all cohorts were normal distributed according to the Shapiro-Wilk-test, otherwise with the Mann-Whitney-U and Kruskal–Wallis test. Significance was assumed if both tests had a significance level of α ≤ 0.05. Furthermore, a receiver operating characteristics (ROC) analysis was performed to evaluate the optimal threshold between the cohorts. The post-hoc standardisation ROC were compared to the intra-scanner-intra-sequence ROC before standardisation to evaluate the maintenance of the diagnostic accuracy. Further, evidence was assumed if the sum of sensitivity and specificity reached 150% or above as recommended in literature.^22^ Finally, intra-subject progression plots were performed with HTE subjects that were measured in different CP environments.

### Implementation

**Fig. 2:**
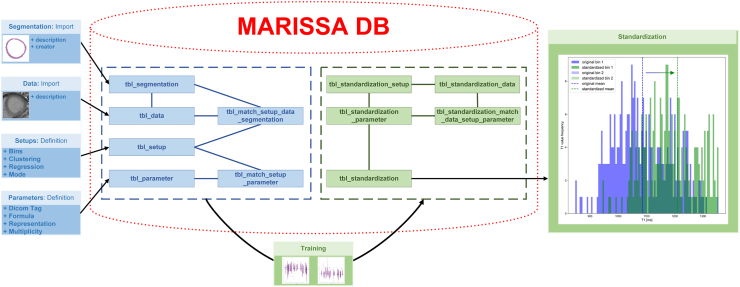
MARISSA structure – The central element is the MARISSA DB based on a SQLite database with relational connected tables: The blue tables tbl_segmentation, tbl_data, tbl_setup, tbl_parameter, tbl_match_setup_data_segmentation and tbl_match_setup_parameter are those, where user interaction takes place by adding, editing or deleting information and data. When starting the training of a standardisation pipeline, the confounding parameter impact estimation is fitted. In order to track back the training, the necessary information is copied into the separate green tables tbl_standardization_setup, tbl_standardization_data, tbl_standardization_parameter and tbl_standardization_match_data_setup_parameter while the tbl_standardization stores the fitted regression models for the confounding parameter impact estimation. Applying the standardisation on a dataset will transform the T1 values into a range that represents the reference confounding parameter environment as depicted on the right site.

The core of MARISSA is a SQLite database in the backend, referred in the following as MARISSA DB, and an overlaying graphical user interface (GUI). Fig. 2 shows abstractly the structure and user interaction in MARISSA. The software was fully implemented in Python (Version 3.8, Python Software Foundation, Beaverton, USA) and is available in the Supplemental Material S2.^23^ All necessary site packages, installation instructions and further detailed information are listed in the MARISSA User Manual in the Supplemental Material S3.

As MARISSA is not only implemented for parametric T1 mapping in CMR, the software works in separate projects with individual MARISSA DBs, that are exportable with or without data. Within MARISSA DB, the related tables are separated: On the one hand the active site, where the user manipulates data, settings and parameters and a passive site that contains all trained standardisation pipelines including a copy of all necessary information to reconstruct the training. This separation assures for a retraceable standardisation pipeline training whereas an export of the project without data loses the traceability while still maintaining the standardisation functionality.

The definition of CPs is based on DICOM tags. Standard DICOM tags are already available in MARISSA, while specific CPs like the sequence variant are extracted by string processing of the series description. The choice of the value representation defines the CP as either a numerical or categorical parameter. MARISSA supports also multi-value DICOM tags as long as the multiplicity remains stable. In this case, each value dimension is considered individually as an own CP. The DICOM standard gives more information about the DICOM tag composition.^24^

The import of DICOM data and segmentations includes a customisable description in order to enable cohort differentiation within the MARISSA DB. While training data must be imported, the standardisation pipeline is applicable on imported as well as external data. Applying the standardisation on a dataset exports the original DICOM data, an Excel table, a MARISSADATA file and a progression plot. The Excel table contains information about the CP values and the transformation of the segmented T1 values while the MARISSADATA file contains the same information as a pickled Python dictionary such that it can be imported and further processed in other Python applications. More detailed information about the usage of MARISSA is provided in the Supplemental Material S3.

### Ethical approval

This study was approved by the local ethics committee of the Charité Universitätsmedizin Berlin as retrospective study (study ID: EA 1253 21) and complies with the declaration of Helsinki. The requirement for written informed consent was acquired during the original clinical studies and was therefore waived in this study due to its retrospective design as approved by the local ethics committee of the Charité Universitätsmedizin Berlin (study ID: EA 1253 21). Due to institutional law, datasets cannot be shared.

### Role of the funding source

This study was supported by the BMBF (Bundesministerium für Bildung und Forschung)/DZHK (German Centre for Cardiovascular Research) via project FKZ81Z0100208. The BMBF/DZHK had no influence on the design, execution or evaluation of this study.

## Results

The results are given for the three steps Confounding Parameters Impact Estimation (CPIE), Best Performing Standardisation Pipeline (BPSP) and Diagnostic Implication (DI) separately as well as the Implementation of MARISSA.

### Confounding Parameters Impact Estimation (CPIE)

The CPIE could be successfully trained without abortion on all 24 settings without and 216 settings with clustering. However, as the individual mode requires constant CP values for all CPs except the estimating one, the training with the used HTR could not include the sequences T1 map MOLLI 3(3)5 b and T1 map SASHA GRE due to variations in the other CPs. Consequently, these two sequences could not be standardised and acted like no bias.

### Best performing standardisation pipeline (BPSP)

The top three settings across the 24 settings without clustering were LSVR regression on relative values in cascaded mode, ETR on relative values in ensemble mode and ETR on absolute values in ensemble mode with a COV of 5.98%, 6.10% and 6.23% respectively for the mean T1 value of the respective standardised HTE. Among all 240 trained pipelines, the BPSP was obtained with the LSVR regression on relative values in the cascaded mode with two bins and the agglomerative single clustering resulting in a COV of 5.81%.

**Fig. 3:**
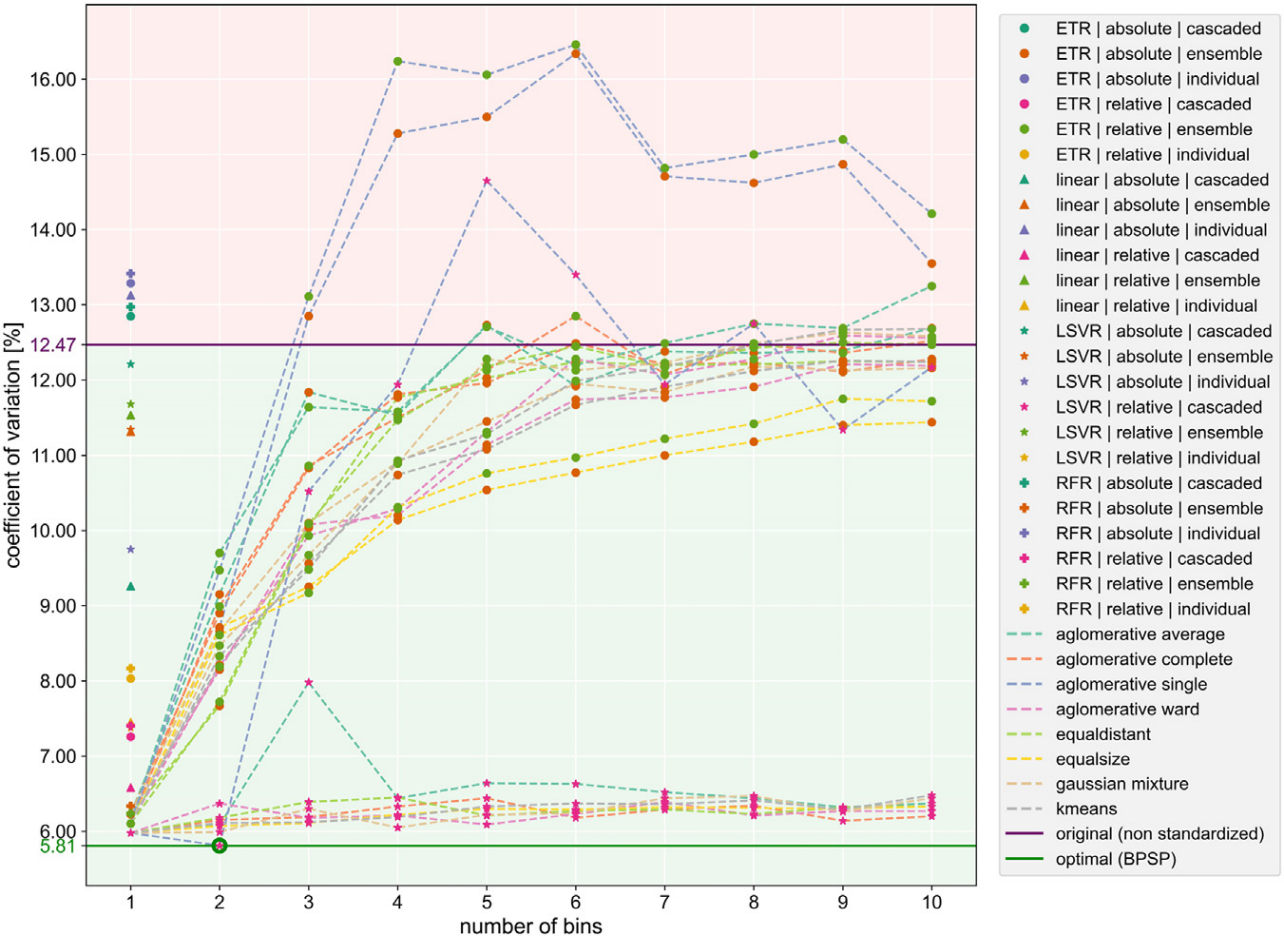
Coefficient of variation (COV) in the 240 trained standardisation pipelines – The COV is plotted against the standardisation pipeline setting denoted as the number of bins on the x-axis, the regression-type by the scatter point marker style, the y-type and mode according to the scatter point colour and the clustering algorithm according to the dotted line for bins greater than one. The purple solid line represents the COV threshold of the unstandardised data with everything above in the red area means a worsening while everything below in the green area means an improvement of the intra-healthy-subjects variation. The green line with the inline circle shows the optimal COV reached among the 240 pipelines representing the best performing standardisation pipeline (BPSP).

Fig. 3 plots the COV for each trained standardisation pipeline including the best obtained COV and the COV of the unstandardised HTE of 12.47% revealing that some standardisation pipeline settings even worsen the uncertainty in the HTE.

### Diagnostic Implication (DI)

**Fig. 4:**
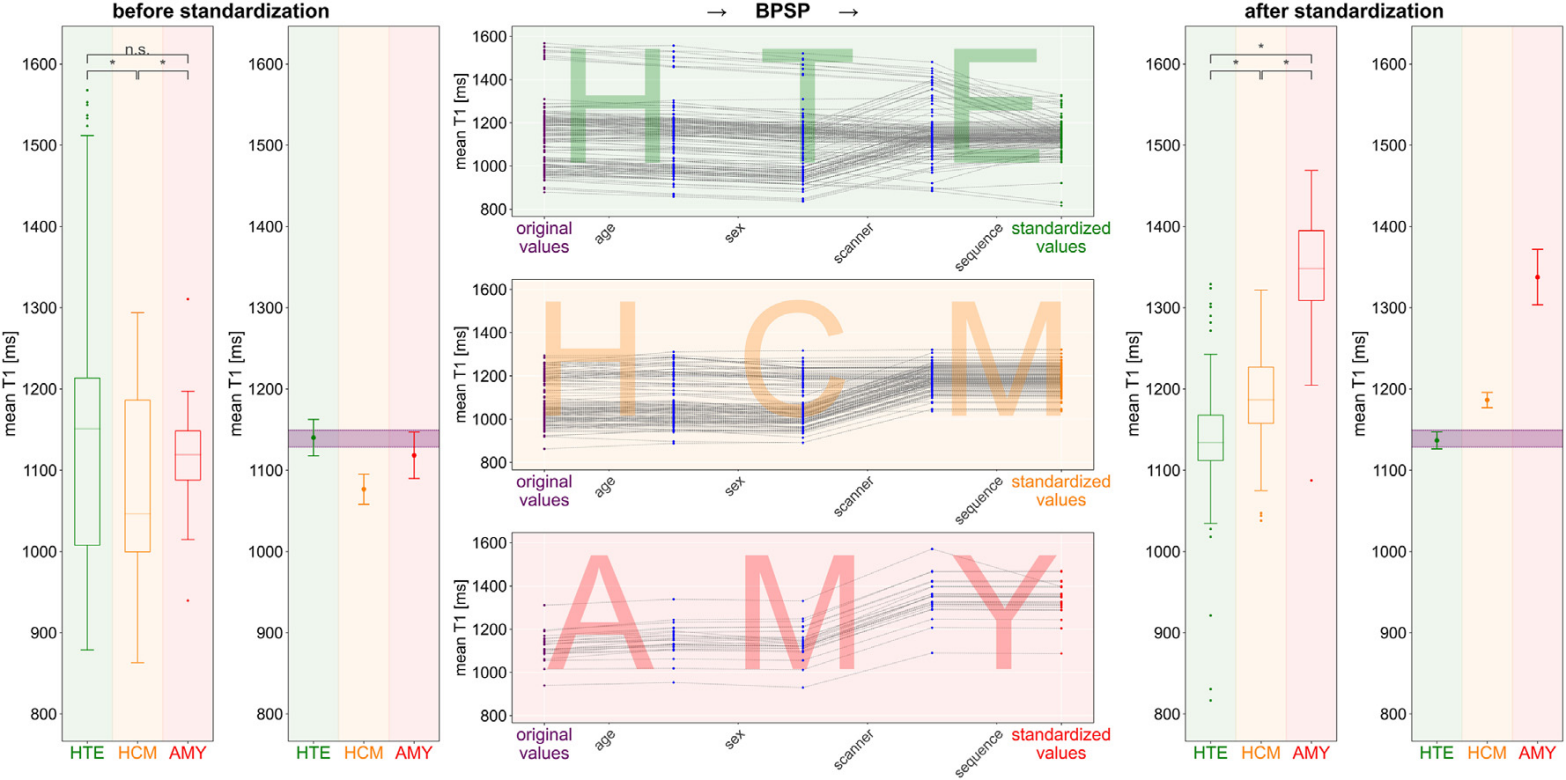
Inter-cohort progression plot – On the left and right are respectively before and after standardisation with the best performing standardisation pipeline (BPSP) the boxplot of the mean T1 value for each cohort: Healthy test datasets (HTE), patients with left ventricular hypertrophy including hypertrophic cardiomyopathy (HCM) and patients with amyloidosis (AMY). The ∗ denotes statistically significant differences and n. s. means not significant. Further, the confidence intervals are plotted against the purple area that represents the confidence interval of the unstandardised healthy data which captures the reference confounding parameter environment. In the middle the progression from original towards standardised values with the BPSP are plotted with detailed impact for each confounding parameter.

Applying the BPSB on the three test cohorts HTE, HCM and AMY results in a COV of 5.81%, 4.46% and 6.05% respectively compared to an unstandardised COV of 12.47%, 9.56% and 6.06%. Hence, COVs for HTE and HCM showed an improvement after standardisation while the COV for AMY remained almost equal. Fig. 4 gives an overview of the respective cohort data before and after standardisation with the BPSP and the individual impact of each CP on every single test dataset.

Already before standardisation the HTE and HCM as well as HCM and AMY but not HTE and AMY were statistically significant different. However, the 25%–75% quantile ranges of HCM (999.57–1186.00 ms) and AMY (1087.84–1148.40 ms) were almost completely within the range of HTE (1007.81–1213.34) and the mean value of both patient cohorts (HCM: 1076.69 ms / AMY: 1118.39 ms) were lower than in HTE (1140.20 ms) while both patient groups are expected with significant higher T1 values.^15^ This contradiction to literature were due to the mixture among CPs in the data collection, especially in regard of the occurring scanner and sequence combinations according to the detailed cohort dataset breakdown in the Supplemental Material S1. After standardisation, all cohorts revealed statistically significant difference from each other and no overlapping CIs. None of the cohorts, neither before nor after standardisation, could remain in the margin of the CI of the unstandardised HTR data, which captured the reference CP environment. However, after standardisation the HTE was closest to fit in whereas HCM and AMY were clearly above. The resulting T1 value ranges (mean ± standard deviation) after standardisation were 1136.78 ± 66.09 ms, 1186.27 ± 52.93 ms and 1337.62 ± 80.92 ms for HTE, HCM and AMY respectively.

**Fig. 5:**
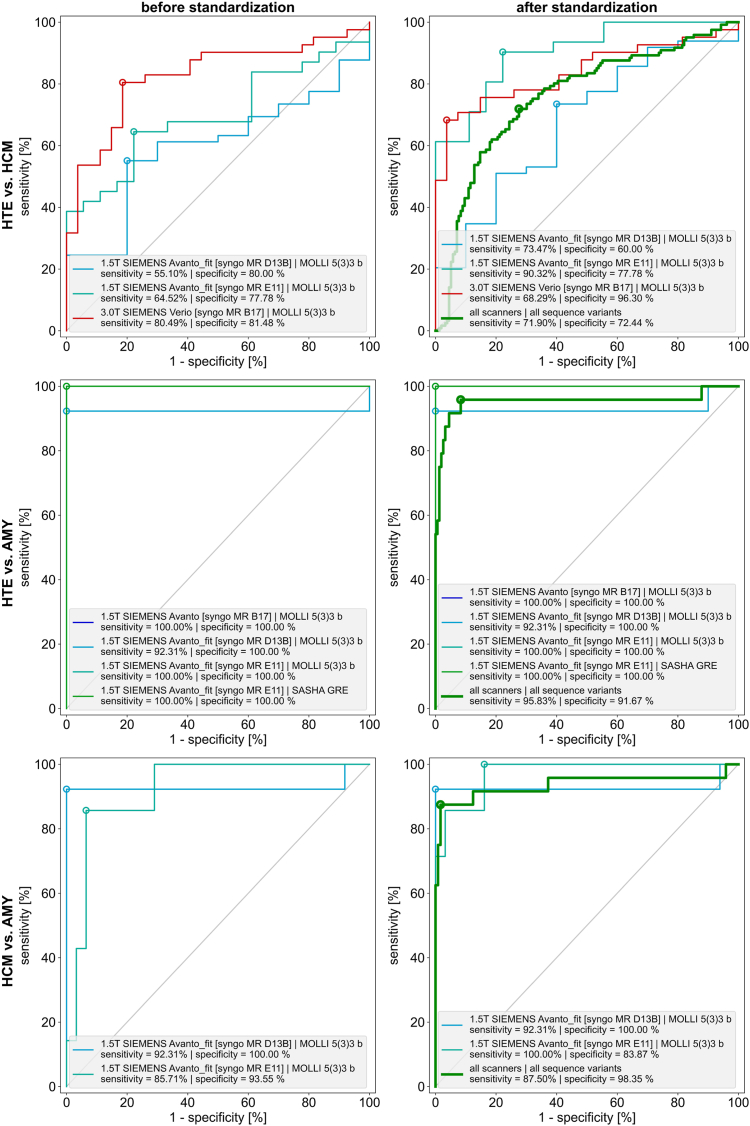
ROC analysis – The left site represents before and the right site after standardisation. Each curve represents intra-scanner-intra-sequence data. After standardisation shows additionally the ROC analysis in a bold green curve that reflects the differentiability among all healthy test datasets (HTE), patients with left ventricular hypertrophy including hypertrophic cardiomyopathy (HCM) and patients with amyloidosis (AMY).

The ROC analysis, as shown in Fig. 5, revealed an optimal threshold (sensitivity / specificity) of 1163.89 ms (71.90% / 72.44%) between HTE and HCM, 1204.46 ms (95.83% / 91.67%) between HTE and AMY and 1287.89 ms (87.50% / 98.35%) between HCM and AMY after standardisation. The differentiation between HTE and HCM were slightly below the 150% threshold for the sum of sensitivity and specificity while HTE and AMY as well as HCM and AMY were above it.^22^ In all three post-hoc standardised ROC analysis sensitivity and specificity were in the range of unstandardised intra-scanner-intra-sequence differentiability. The sum of sensitivity and specificity increased or at least remained after standardisation within the intra-scanner-intra-sequence datasets although individual sensitivity and specificity values changed.

**Fig. 6:**
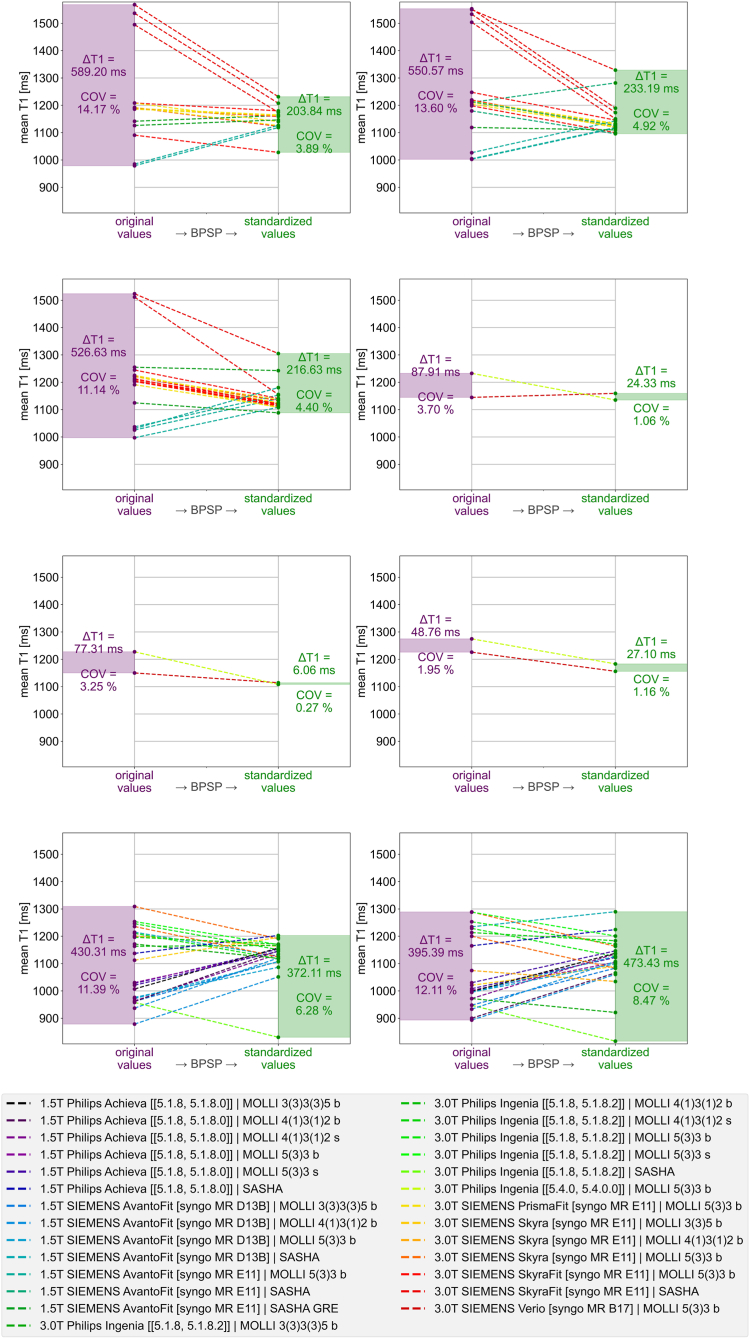
Intra-subject progression plot – Eight healthy volunteers underwent at least two different acquisitions. The respective standardisation with the best performing standardisation pipeline (BPSP) is shown for each scanner and sequence combination. The violet bar on the left of each plot shows the value spread before and the green bar on the right the value spread after standardisation as well as the coefficient of variation (COV).

Finally, eight subjects of the HTE group received at least two different acquisitions. The intra-subject progression plot for each of these subjects is shown in Fig. 6. The plot shows a minimisation of the value spread after standardisation in all subjects but one. Nonetheless, all subjects showed a minimisation of the COV, which reflects a concentration of the acquisitions after standardisation. However, individual measurements, especially SASHA based parametric T1 maps, revealed high imprecision after standardisation.

### Implementation

The MARISSA was successfully implemented as a Python software tool with a SQLite database backend. The overlaying GUI allows usability for programmers and clinicians alike. The definition of custom CPs, however, requires at least experience in the DICOM tag standard as well as Python string processing. Furthermore, data filtering in the GUI works by SQL commands via respective input fields. The software enables to work in projects that can be exported with and without data.

## Discussion

In this work, we were able to show that a post-hoc standardisation of parametric T1 maps in CMR is feasible while maintaining disease differentiability according to sensitivity and specificity as in intra-scanner-intra-sequence scenarios. We could further show that the choice of the standardisation pipeline settings was crucial for the overall performance. The considered CPs of age, sex, scanner and sequence revealed the LSVR regression on relative values in the cascaded mode with two bins and the agglomerative single clustering as the BPSP among 240 tested standardisation pipelines with respect to the HTE of native, mid-ventricular parametric T1 maps in CMR. The implemented MARISSA including a GUI is made available with this work for further development and evaluation. In the following, a detailed discussion about the Dataset, the results of Confounding Parameters Impact Estimation (CPIE), Best Performing Standardisation Pipeline (BPSP), Diagnostic Implication (DI) and Implementation is presented and ends up with the Limitations and a short Conclusion.

### Dataset

The included datasets were retrospectively collected from available data with a diagnosis of either Healthy, HCM or AMY. The data were not filtered except for native, artifact-free, midventricular slices. Consequently, the data were not balanced according to scanner-sequence combinations. The examination of both patient cohorts was mainly performed on 1.5T Siemens scanners with less sequence variations resulting in, contrary to literature,^15^ lower average T1 values than HTE before standardisation. Other established manufacturers were not included due to missing access within the Berlin CMR research network.^10^ The considered patient cohorts HCM and AMY reflect only two CVDs with expected significant higher T1 values. The diagnostic performance on other, more subtle, CVDs requires future investigation.

Regarding the segmentation, different strategies exist.^11^^,^^12^ While the segmentation of the septum is more precise,^4^ it lacks the majority of the myocardial voxels. Therefore, this work used a full circular segmentation of the myocardium at the cost of an increasing standard deviation in the T1 values compared to septal segmentation only.^12^

### Confounding Parameters Impact Estimation (CPIE)

In this work, the CPIE trained regression models to estimate the bias between apparent and target T1 values based on a healthy volunteer cohort. Subjects with CVDs were excluded in this step, as those would have an unintentional influence towards either higher or lower T1 values.^25^ The CPIE, as central part of the post-hoc standardisation pipeline, enables the comparability of parametric T1 mapping. Although multiple studies were published on reference values for parametric T1 mapping over the last decade, those were only valid in a specific cohort and technical setting.^12^^,^^26^^,^^27^

The z-Score transformation into a unitless value domain enabled as the solely established approach the comparability across different CP environments.^28^ However, the z-Score calculation is based on a local healthy reference cohort. Consequently, a healthy cohort examination is not only performed after initial operation but always necessary after a hard- or software change of the MR scanner that perturbs the T1 value distribution of the healthy cohort.^28^ Although considered in the current guidelines,^4^ this obstacle of additional effort and costs circumvent the establishment of the z-Score in the clinical routine. The inclusion of novel CP values or even CPs themselves requires, likewise the z-Score transformation, additional healthy volunteer examinations. However, thanks to the transfer learning capability of MARISSA, single site scans are sufficient to apply CP value standardisation on other sites. Consequently, the amount of healthy volunteer examinations and thereby the cost may be reduced with our standardisation approach compared to the z-Score. Nonetheless, an increasing amount of training data is necessary over time, which is limited by the accessibility and potential restrictions due to institutional or governmental law.

The number of necessary training data highly depends on the standardisation pipeline setting, especially the mode. The individual mode already revealed in this work, that two sequences could not be captured during training as all other CPs were expected to be constant. Hence, the individual mode is prone to the training data and most likely misses certain CP values. However, it allows for the best isolation of CP's influence. Compared to that, the cascaded mode depends on the order of the CPs as the first one works the same way as the individual mode while the last one can consider the whole training dataset as all other CPs are already standardised. As a consequence, inter-parameter correlations are partly considered, but some CP values might be missed as well if the training dataset or the order of the considered CPs is not well chosen. Finally, the ensemble mode considers all in one and catches all CP values that were given in the training data. The ensemble mode accounts for inter-parameter correlation best, but fails completely if a test dataset includes a categorical value that was not in the training dataset. The individual and cascaded mode on the contrary still standardise for all other CPs and can skip those that are unknown. As a rule of thumb, the number of necessary training data increases from ensemble to cascaded to individual mode in order to capture all CP values.

As this study is a proof-of-concept for a standardisation approach of parametric T1 maps in CMR, only a limited number of parameters were included. There are other parameters such as heart rate (HR),^9^^,^^29^ the body-mass-index (BMI)^30^ or the voxel size^31^ that have a known relevant impact. However, BMI and HR could not be included as the necessary information were not available in all datasets of this study due to data anonymisation and its retrospective character. Further, all three CPs are numerical variables, resulting in a much higher necessary amount of training data for the individual and cascaded mode. On the contrary, each numerical variable can be turned into a categorical one by clustering, for example the age can be divided into decades or the BMI into the groups proposed by the world health organization.^32^

The patient specific parameters age and sex were included as potential CPs, but showed an ambivalent impact in the literature. Roy et al.^11^ revealed a significant whereas Dabir et al.^12^ showed only a slight but not significant impact due to age and sex. Our results, in turn, emphasised a non-zero induced bias, however, much less than the scanner or sequence originated biases. If age and sex turn into neglectable CPs compared to other potential CPs, those may be excluded easily from the standardisation pipeline within MARISSA in the future.

The considered CPs in this work treated subject specific and technological variations only. However, post-processing procedure variations represent CPs in parametric T1 mapping as well.^8^ The post processing comprises everything from image reconstruction^8^ to segmentation procedures.^33^ While reconstruction algorithms are not available from DICOM tags, the segmentation method of the myocardium is still based on expert agreement or in accordance to guidelines.^34^ Although fully automated segmentation procedures exist,^16^ manual adaptions are still necessary. Consequently, the segmentation method is currently a local specific, hard to definable CP. In this work, the segmentation was performed according to a pre-defined standard operating procedure. However, in future perspective, when including more training data, this must be considered, especially since artificial intelligence assisted segmentation procedures become ever more popular.^16^^,^^35^

### Best performing standardisation pipeline (BPSP)

As the post-hoc standardisation pipeline can be computed in different settings, the results showed that the setting choice is crucial for the performance of the pipeline with respect to the COV as quality index. Some pipeline settings even showed a worsening of the COV compared to the unstandardised values assuming a non-suitable standardisation pipeline setting. Although the BPSP among 240 evaluated standardisation pipeline settings included a clustering into two bins, the performance gain of 0.17% due to the clustering was rather small compared to a COV reduction of up to 6.49% by a standardisation pipeline without clustering. In most cases the clustering even worsened the outcome. Therefore, the determination of the suitable regression-type, y-type and mode is most important whereas clustering into bins reflects a rather potential fine-tuning step.

### Diagnostic Implication (DI)

The BPSP allowed for a statistically significant differentiation of the three cohorts: HTE, HCM and AMY. However, the sum of sensitivity and specificity across HTE and HCM was below 150% due to a high overlap and thus not sufficient for evidence according to literature.^22^ This aligns with literature values on 3T scanners for Healthy and HCM that show also a significant difference but high overlap as in Liang et al. (1228.4 ± 42.7 ms vs. 1290.0 ± 64.3 ms),^36^ Qin et al. (1240.0 ± 29.8 ms vs. 1308.0 ± 55.5 ms)^37^ and Lavall et al. (1225 ± 21 ms vs. 1266 ± 44 ms).^15^ However, as HCM has manifold morphologies due to a large variety of genotypes and risk factors, the disease state changes over time, which in turn affects the amount of diseased myocardial tissue.^38^^,^^39^

Baggiano et al.^40^ showed a sensitivity of 85% and specificity of 87% when comparing Healthy with AMY that could be outperformed with our BPSP with a sensitivity of 95.83% and specificity of 91.67%. However, they were able to include 436 patients with amyloidosis, which naturally assumes a higher value spread in that patient cohort compared to our 24 included ones. The z-Score approach reached in the study by Kranzusch et al. an equivalent sensitivity of 96% but an improved specificity of 100%.^28^

In the discrimination of both patient groups, HCM and AMY, the sensitivity (87.5%) and specificity (98.35%) were in range of published literature values by Lavall et al. (100% / 97%),^15^ Nam et al. (76.1% / 83.3%)^41^ and Martinez-Naharro (86.54% / 80.36%).^42^ Consequently, amyloidosis is reliably detectable after standardisation while HCM only in an advanced state.

When going from the global cohort perspective into the intra-subject view, the BPSP managed to decrease the COV within the same subject across different acquisitions. Although most acquisitions could be harmonised towards equal values, individual outliers remained after standardisation. Those outliers originated mainly either from a SASHA based sequence, which are assumed to have a higher accuracy but lower precision than MOLLI based sequences,^9^ or already had unusual values for the specific field strength and sequence scheme setup. This, however, shows the limits of MARISSA pipelines. On the one hand, outlying or unusual values will remain outlying or unusual after standardisation and, on the other hand, imprecision cannot be improved. Consequently, the used data for training and testing need a high degree of precision. This does not only affect the used sequence variant but also demands highly controlled production process of the magnetic resonance imaging scanner. High tolerances undermine the generalisability of the proposed post-hoc standardisation pipeline approach. This susceptibility to imprecision is also shared by the z-Score approach whose usability is undermined by high fluctuations in the standard deviation.^43^ The standard deviation of measured T1 values can be minimised by solely segmenting the septal region^12^ instead of the full midventricular myocardium as performed in this work. However, this segmentation strategy would miss the majority of the myocardial tissue.

Finally, it is important to mention, that the standardisation approach calculates the impact of a CP compared to a reference CP value. Consequently, the standardised T1 maps become comparable, but do not necessarily represent the true T1 relaxation value of the myocardial tissue. The reference sequence MOLLI 5(3)3 b for example is known to underestimate the true T1 value.^9^

### Implementation

As MARISSA was fully implemented in Python, it can be installed and run on all major operating systems. Export functionality enables the sharing of trained standardisation pipelines either with or without data. Although tested for parametric T1 maps of the heart only, MARISSA is intended for usability among other quantitative methods, like parametric T2 maps,^44^ or tissues, such as the liver.^45^

Furthermore, MARISSA is extensible in the future. Additional conceivable CPs can be entered into MARISSA via the GUI while novel clustering algorithms and regression models are easily implementable due to a standardised structure.

A subsequent development of MARISSA may include further adjustment options within the GUI. This comprises for example the hyperparameter setup of regression models and clustering algorithms beyond the standard setting or individual settings for each CP within a standardisation pipeline.

### Limitations

The major limitation of this work is the unbalanced underlying dataset due to its retrospective design. The lack of further scanner-sequence combinations limits the generalisability of the proposed standardisation pipeline. The transfer learning capability of scanner-sequence combinations that are not reflected in the training data but captured by the BPSP requires further investigation. This work only contained two cases in the AMY cohort whose scanner-sequence combination were not reflected in the training data. Additionally, a more convincing DI requires more scanner-sequence variability in the considered patient groups. As this work is a proof-of-concept and includes anonymised data, further known relevant CPs were not included and should be considered in a future state. The inclusion of mid-ventricular slices only does not meet all manifold phenotypes of an HCM which may affect only various local regions rather than the whole ventricle.^46^

### Conclusion

All in all, we were able to introduce the MARISSA to enable post-hoc standardisation pipelines for parametric T1 mapping in CMR. The diagnostic power after standardisation with the BPSP in our proof-of-concept were equivalent to those found in literature. The performance of the standardisation pipeline highly depends on the pipeline setting and the precision of the provided data. The current results give hope to improve comparability when adding more training data and considered CPs in the future.

## Contributors

Darian Viezzer – Conceptualisation, Methodology, Data Curation, Formal Analysis, Project Administration, Software, Validation, Visualisation, Writing (original draft).

Thomas Hadler – Methodology, Formal Analysis, Software, Validation, Writing (review & editing).

Jan Gröschel – Data Curation, Formal Analysis, Writing (review & editing).

Clemens Ammann – Methodology, Software, Writing (review & editing).

Edyta Blaszczyk – Data Curation, Formal Analysis, Writing (review & editing).

Christoph Kolbitsch – Methodology, Writing (review & editing).

Simone Hufnagel – Data Curation, Writing (review & editing).

Riccardo Kranzusch-Groß – Data Curation, Writing (review & editing).

Steffen Lange – Conceptualisation, Methodology, Formal Analysis, Supervision, Writing (review & editing).

Jeanette Schulz-Menger – Conceptualisation, Data Curation, Formal Analysis, Supervision, Writing (review & editing).

All authors read and approved the final version of the manuscript and ensured it is the case. Darian Viezzer, Jan Gröschel and Edyta Blaszczyk accessed and verified the complete underlying data.

## Data sharing statement

The trained standardisation pipelines are available on reasonable request by contacting the first (DV: darian-steven.viezzer@charite.de) or last (JSM: jeanette.schulz-menger@charite.de) author. Due to institutional law, datasets cannot be shared. The source code can be accessed via GitHub under the URL: https://github.com/DSV-CUB/MARISSA or in the Supplemental Material S2.^23^

## Declaration of interests

The authors declare no competing interests and no usage of AI or AI assisted technologies for the scientific writing.

## Appendix A Supplementary data

S1 - Detailed Dataset Breakdown

S2 - MARISSA Source Code

S3 - MARISSA User Manual
