# Supplementary material for: Post-hoc standardisation of parametric T1 maps in cardiovascular magnetic resonance imaging: a proof-of-concept
Source: eBioMedicine. 2024 Mar 14;102:105055. doi: 10.1016/j.ebiom.2024.105055 (PMC10951905; doi:10.1016/j.ebiom.2024.105055)
Supplement: S1 - Detailed Dataset Breakdown [file mmc1.pdf]

S1.1: Healthy cohort details with N=number of subjects, M=number of T1 maps, Nm=number of male subjects, Mm=number of male T1 maps, T1m=T1 time of male subjects [ms], am=age of male subjects [years], Nf=number of female subjects, Mf=number of female T1 maps, T1f=T1 time of female subjects [ms], af=age of female subjects [years], T1 values and age are given as mean±standard deviation, green denotes scanner-sequence-combinations represented in the training dataset

**N=254**<br>**M=970**	1.5T				3.0T						
	Philips<br>Achieva<br>[5.1.8.0]	Siemens<br>Avanto<br>[syngo MR B17]	Siemens<br>AvantoFit<br>[syngo MR D13B]	Siemens<br>AvantoFit<br>[syngo MR E11]	Philips<br>Ingenia<br>[5.1.8.2]	Philips<br>Ingenia<br>[5.4.0.0]	Philips<br>Ingenia<br>[5.4.0.1]	Siemens<br>PrismaFit<br>[syngo MR E11]	Siemens<br>Skyra<br>[syngo MR E11]	Siemens<br>SkyraFit<br>[syngo MR E11]	Siemens<br>Verio<br>[syngo MR B17]
MOLLI<br>3(3)3(3)5 b	N=16 | M=16<br>Nm=8 | Mm=8<br>T1m=976.07±27.25<br>am=24.88±3.98<br>Nf=8 | Mf=8<br>T1f=1009.98±16.47<br>af=24.62±3.43		N=16 | M=17<br>Nm=7 | Mm=7<br>T1m=967.35±40.32<br>am=25.14±4.19<br>Nf=9 | Mf=10<br>T1f=951.54±24.93<br>af=24.44±3.27		N=15 | M=17<br>Nm=8 | Mm=10<br>T1m=1118.68±85.44<br>am=24.88±3.98<br>Nf=7 | Mf=7<br>T1f=1107.83±94.29<br>af=25.00±3.51						N=14 | M=18<br>Nm=12 | Mm=16<br>T1m=1239.78±18.15<br>am=31.50±6.85<br>Nf=2 | Mf=2<br>T1f=1273.78±29.29<br>af=31.00±2.00
MOLLI<br>3(3)5 b									N=16 | M=16<br>Nm=8 | Mm=8<br>T1m=1054.72±44.71<br>am=25.00±3.94<br>Nf=8 | Mf=8<br>T1f=1094.66±42.35<br>af=24.62±3.43		
MOLLI<br>4(1)3(1)2 b	N=16 | M=16<br>Nm=8 | Mm=8<br>T1m=931.07±26.66<br>am=24.88±3.98<br>Nf=8 | Mf=8<br>T1f=933.28±32.50<br>af=24.62±3.43		N=16 | M=32<br>Nm=7 | Mm=14<br>T1m=931.84±36.48<br>am=25.14±4.19<br>Nf=9 | Mf=18<br>T1f=928.80±29.56<br>af=24.44±3.27		N=9 | M=9<br>Nm=4 | Mm=4<br>T1m=1137.29±129.28<br>am=25.50±5.50<br>Nf=5 | Mf=5<br>T1f=1155.10±34.22<br>af=25.40±3.20				N=16 | M=17<br>Nm=8 | Mm=9<br>T1m=1103.06±51.08<br>am=25.00±3.94<br>Nf=8 | Mf=8<br>T1f=1188.99±81.84<br>af=24.62±3.43		
MOLLI<br>4(1)3(1)2 s	N=16 | M=16<br>Nm=8 | Mm=8<br>T1m=928.76±25.50<br>am=24.88±3.98<br>Nf=8 | Mf=8<br>T1f=955.14±17.82<br>af=24.62±3.43				N=12 | M=13<br>Nm=5 | Mm=6<br>T1m=1173.27±64.45<br>am=25.20±4.96<br>Nf=7 | Mf=7<br>T1f=1190.78±34.45<br>af=25.00±3.51						
MOLLI<br>5(3)3 b	N=16 | M=16<br>Nm=8 | Mm=8<br>T1m=994.97±27.72<br>am=24.88±3.98<br>Nf=8 | Mf=8<br>T1f=1009.58±14.07<br>af=24.62±3.43	N=8 | M=8<br>Nm=5 | Mm=5<br>T1m=960.47±20.67<br>am=25.20±3.76<br>Nf=3 | Mf=3<br>T1f=1027.73±72.36<br>af=36.00±13.74	N=44 | M=62<br>Nm=20 | Mm=28<br>T1m=998.41±38.24<br>am=37.20±16.14<br>Nf=24 | Mf=34<br>T1f=1002.36±31.67<br>af=43.21±16.86	N=57 | M=100<br>Nm=30 | Mm=50<br>T1m=972.42±18.89<br>am=33.07±10.90<br>Nf=27 | Mf=50<br>T1f=1015.45±28.44<br>af=36.56±12.89	N=15 | M=30<br>Nm=8 | Mm=17<br>T1m=1171.66±88.13<br>am=24.88±3.98<br>Nf=7 | Mf=13<br>T1f=1240.45±23.86<br>af=25.00±3.51	N=15 | M=15<br>Nm=7 | Mm=7<br>T1m=1214.04±27.86<br>am=24.29±6.96<br>Nf=8 | Mf=8<br>T1f=1243.49±17.87<br>af=24.50±3.46	N=2 | M=2<br>Nm=1 | Mm=1<br>T1m=1209.17±0.00<br>am=24.00±0.00<br>Nf=1 | Mf=1<br>T1f=1231.24±0.00<br>af=24.00±0.00	N=17 | M=78<br>Nm=10 | Mm=50<br>T1m=1191.82±25.12<br>am=26.50±3.32<br>Nf=7 | Mf=28<br>T1f=1217.93±35.03<br>af=25.57±8.43	N=16 | M=32<br>Nm=8 | Mm=16<br>T1m=1217.11±53.17<br>am=25.00±3.94<br>Nf=8 | Mf=16<br>T1f=1267.37±41.26<br>af=24.62±3.43	N=21 | M=74<br>Nm=14 | Mm=43<br>T1m=1187.96±29.83<br>am=29.07±6.69<br>Nf=7 | Mf=31<br>T1f=1226.43±26.89<br>af=25.57±8.43	N=93 | M=172<br>Nm=44 | Mm=72<br>T1m=1139.24±39.42<br>am=45.23±17.41<br>Nf=49 | Mf=100<br>T1f=1167.97±34.79<br>af=45.29±16.77
MOLLI<br>5(3)3 s	N=16 | M=32<br>Nm=8 | Mm=16<br>T1m=982.84±25.58<br>am=24.88±3.98<br>Nf=8 | Mf=16<br>T1f=1013.30±22.42<br>af=24.62±3.43				N=15 | M=15<br>Nm=8 | Mm=8<br>T1m=1195.00±99.65<br>am=24.88±3.98<br>Nf=7 | Mf=7<br>T1f=1262.68±20.61<br>af=25.00±3.51						N=31 | M=31<br>Nm=0 | Mm=0<br>T1m=0.00±0.00<br>am=0.00±0.00<br>Nf=31 | Mf=31<br>T1f=1084.04±41.49<br>af=30.81±5.32
SASHA	N=13 | M=13<br>Nm=6 | Mm=6<br>T1m=1023.18±96.71<br>am=23.17±1.07<br>Nf=7 | Mf=7<br>T1f=1160.07±42.06<br>af=24.14±3.40		N=16 | M=16<br>Nm=7 | Mm=7<br>T1m=1212.80±35.72<br>am=25.14±4.19<br>Nf=9 | Mf=9<br>T1f=1249.89±32.37<br>af=24.44±3.27	N=6 | M=12<br>Nm=4 | Mm=7<br>T1m=1140.82±30.06<br>am=28.25±3.27<br>Nf=2 | Mf=5<br>T1f=1212.32±18.12<br>af=23.00±1.00	N=10 | M=10<br>Nm=4 | Mm=4<br>T1m=797.43±51.18<br>am=23.25±1.30<br>Nf=6 | Mf=6<br>T1f=1184.99±175.16<br>af=25.17±3.76					N=17 | M=56<br>Nm=10 | Mm=32<br>T1m=1498.43±31.83<br>am=26.90±3.48<br>Nf=7 | Mf=24<br>T1f=1536.87±47.16<br>af=25.57±8.43	
SASHA GRE				N=7 | M=9<br>Nm=4 | Mm=5<br>T1m=1124.40±41.99<br>am=28.00±4.06<br>Nf=3 | Mf=4<br>T1f=1187.34±65.67<br>af=22.67±2.49							

S1.2: Healthy train cohort (HTR) details with N=number of subjects, M=number of T1 maps, Nm=number of male subjects, Mm=number of male T1 maps, T1m=T1 time of male subjects [ms], am=age of male subjects [years], Nf=number of female subjects, Mf=number of female T1 maps, T1f=T1 time of female subjects [ms], af=age of female subjects [years], T1 values and age are given as mean±standard deviation, green denotes scanner-sequence-combinations represented in the training dataset

<table>
<tr><th rowspan="2">N=214
M=814</th><th colspan="4">1.5T</th><th colspan="7">3.0T</th></tr>
<tr><th>Philips
Achieva
[5.1.8.0]</th><th>Siemens
Avanto
[syngo MR B17]</th><th>Siemens
AvantoFit
[syngo MR D13B]</th><th>Siemens
AvantoFit
[syngo MR E11]</th><th>Philips
Ingenia
[5.1.8.2]</th><th>Philips
Ingenia
[5.4.0.0]</th><th>Philips
Ingenia
[5.4.0.1]</th><th>Siemens
PrismaFit
[syngo MR E11]</th><th>Siemens
Skyra
[syngo MR E11]</th><th>Siemens
SkyraFit
[syngo MR E11]</th><th>Siemens
Verio
[syngo MR B17]</th></tr>
<tr><td>MOLLI
3(3)3(3)5 b</td><td>N=14 | M=14
Nm=8 | Mm=8
T1m=976.07±27.25
am=24.88±3.98
Nf=6 | Mf=6
T1f=1012.53±18.13
af=24.67±2.98</td><td></td><td>N=14 | M=15
Nm=7 | Mm=7
T1m=967.35±40.32
am=25.14±4.19
Nf=7 | Mf=8
T1f=950.68±27.59
af=24.43±2.82</td><td></td><td>N=13 | M=15
Nm=8 | Mm=10
T1m=1118.68±85.44
am=24.88±3.98
Nf=5 | Mf=5
T1f=1122.33±87.96
af=25.20±2.99</td><td></td><td></td><td></td><td></td><td></td><td>N=12 | M=14
Nm=10 | Mm=12
T1m=1235.09±15.08
am=29.40±2.37
Nf=2 | Mf=2
T1f=1273.78±29.29
af=31.00±2.00</td></tr>
<tr><td>MOLLI
3(3)5 b</td><td></td><td></td><td></td><td></td><td></td><td></td><td></td><td></td><td>N=14 | M=14
Nm=8 | Mm=8
T1m=1054.72±44.71
am=25.00±3.94
Nf=6 | Mf=6
T1f=1104.07±36.58
af=24.67±2.98</td><td></td><td></td></tr>
<tr><td>MOLLI
4(1)3(1)2 b</td><td>N=14 | M=14
Nm=8 | Mm=8
T1m=931.07±26.66
am=24.88±3.98
Nf=6 | Mf=6
T1f=934.18±33.13
af=24.67±2.98</td><td></td><td>N=14 | M=28
Nm=7 | Mm=14
T1m=931.84±36.48
am=25.14±4.19
Nf=7 | Mf=14
T1f=934.00±28.67
af=24.43±2.82</td><td></td><td>N=8 | M=8
Nm=4 | Mm=4
T1m=1137.29±129.28
am=25.50±5.50
Nf=4 | Mf=4
T1f=1153.09±38.00
af=24.50±2.96</td><td></td><td></td><td></td><td>N=14 | M=15
Nm=8 | Mm=9
T1m=1103.06±51.08
am=25.00±3.94
Nf=6 | Mf=6
T1f=1206.16±80.18
af=24.67±2.98</td><td></td><td></td></tr>
<tr><td>MOLLI
4(1)3(1)2 s</td><td>N=14 | M=14
Nm=8 | Mm=8
T1m=928.76±25.50
am=24.88±3.98
Nf=6 | Mf=6
T1f=950.99±18.67
af=24.67±2.98</td><td></td><td></td><td></td><td>N=10 | M=11
Nm=5 | Mm=6
T1m=1173.27±64.45
am=25.20±4.96
Nf=5 | Mf=5
T1f=1185.21±39.03
af=25.20±2.99</td><td></td><td></td><td></td><td></td><td></td><td></td></tr>
<tr><td>MOLLI
5(3)3 b</td><td>N=14 | M=14
Nm=8 | Mm=8
T1m=994.97±27.72
am=24.88±3.98
Nf=6 | Mf=6
T1f=1007.36±13.34
af=24.67±2.98</td><td>N=7 | M=7
Nm=5 | Mm=5
T1m=960.47±20.67
am=25.20±3.76
Nf=2 | Mf=2
T1f=1056.52±73.27
af=42.50±12.50</td><td>N=37 | M=52
Nm=17 | Mm=25
T1m=1000.38±39.40
am=36.65±16.41
Nf=20 | Mf=27
T1f=1002.97±32.88
af=44.05±16.57</td><td>N=47 | M=82
Nm=24 | Mm=43
T1m=972.15±19.59
am=32.08±9.73
Nf=23 | Mf=39
T1f=1017.25±30.23
af=37.09±12.83</td><td>N=13 | M=26
Nm=8 | Mm=17
T1m=1171.66±88.13
am=24.88±3.98
Nf=5 | Mf=9
T1f=1243.05±26.05
af=25.20±2.99</td><td>N=12 | M=12
Nm=6 | Mm=6
T1m=1210.95±28.96
am=24.83±7.38
Nf=6 | Mf=6
T1f=1241.01±14.64
af=25.50±3.45</td><td>N=2 | M=2
Nm=1 | Mm=1
T1m=1209.17±0.00
am=24.00±0.00
Nf=1 | Mf=1
T1f=1231.24±0.00
af=24.00±0.00</td><td>N=14 | M=65
Nm=9 | Mm=46
T1m=1191.67±26.06
am=26.11±3.28
Nf=5 | Mf=19
T1f=1221.07±41.66
af=27.40±9.35</td><td>N=14 | M=28
Nm=8 | Mm=16
T1m=1217.11±53.17
am=25.00±3.94
Nf=6 | Mf=12
T1f=1270.36±40.19
af=24.67±2.98</td><td>N=18 | M=59
Nm=13 | Mm=39
T1m=1189.87±26.89
am=29.00±6.94
Nf=5 | Mf=20
T1f=1230.81±30.69
af=27.40±9.35</td><td>N=78 | M=145
Nm=37 | Mm=61
T1m=1135.78±39.99
am=44.86±17.38
Nf=41 | Mf=84
T1f=1171.19±33.39
af=45.41±16.59</td></tr>
<tr><td>MOLLI
5(3)3 s</td><td>N=14 | M=28
Nm=8 | Mm=16
T1m=982.84±25.58
am=24.88±3.98
Nf=6 | Mf=12
T1f=1010.58±24.83
af=24.67±2.98</td><td></td><td></td><td></td><td>N=13 | M=13
Nm=8 | Mm=8
T1m=1195.00±99.65
am=24.88±3.98
Nf=5 | Mf=5
T1f=1259.32±20.85
af=25.20±2.99</td><td></td><td></td><td></td><td></td><td></td><td>N=26 | M=26
Nm=0 | Mm=0
T1m=0.00±0.00
am=0.00±0.00
Nf=26 | Mf=26
T1f=1082.55±43.91
af=30.77±5.67</td></tr>
<tr><td>SASHA</td><td>N=11 | M=11
Nm=6 | Mm=6
T1m=1023.18±96.71
am=23.17±1.07
Nf=5 | Mf=5
T1f=1163.70±48.50
af=24.00±2.83</td><td></td><td>N=14 | M=14
Nm=7 | Mm=7
T1m=1212.80±35.72
am=25.14±4.19
Nf=7 | Mf=7
T1f=1257.09±32.95
af=24.43±2.82</td><td>N=5 | M=9
Nm=4 | Mm=7
T1m=1140.82±30.06
am=28.25±3.27
Nf=1 | Mf=2
T1f=1228.91±0.70
af=24.00±0.00</td><td>N=8 | M=8
Nm=4 | Mm=4
T1m=797.43±51.18
am=23.25±1.30
Nf=4 | Mf=4
T1f=1301.14±74.38
af=25.50±3.28</td><td></td><td></td><td></td><td></td><td>N=14 | M=47
Nm=9 | Mm=29
T1m=1494.84±29.79
am=26.56±3.50
Nf=5 | Mf=18
T1f=1539.43±53.18
af=27.40±9.35</td><td></td></tr>
<tr><td>SASHA GRE</td><td></td><td></td><td></td><td>N=4 | M=4
Nm=3 | Mm=3
T1m=1118.18±52.93
am=27.33±4.50
Nf=1 | Mf=1
T1f=1251.05±0.00
af=26.00±0.00</td><td></td><td></td><td></td><td></td><td></td><td></td><td></td></tr>
</table>

S1.3: Healthy test cohort (HTE) details with N=number of subjects, M=number of T1 maps, Nm=number of male subjects, Mm=number of male T1 maps, T1m=T1 time of male subjects [ms], am=age of male subjects [years], Nf=number of female subjects, Mf=number of female T1 maps, T1f=T1 time of female subjects [ms], af=age of female subjects [years], T1 values and age are given as mean±standard deviation, green denotes scanner-sequence-combinations represented in the training dataset

**N=40**<br>**M=156**	1.5T				3.0T						
	Philips<br>Achieva<br>[5.1.8.0]	Siemens<br>Avanto<br>[syngo MR B17]	Siemens<br>AvantoFit<br>[syngo MR D13B]	Siemens<br>AvantoFit<br>[syngo MR E11]	Philips<br>Ingenia<br>[5.1.8.2]	Philips<br>Ingenia<br>[5.4.0.0]	Philips<br>Ingenia<br>[5.4.0.1]	Siemens<br>PrismaFit<br>[syngo MR E11]	Siemens<br>Skyra<br>[syngo MR E11]	Siemens<br>SkyraFit<br>[syngo MR E11]	Siemens<br>Verio<br>[syngo MR B17]
MOLLI<br>3(3)3(3)5 b	N=2 | M=2<br>Nm=0 | Mm=0<br>T1m=0.00±0.00<br>am=0.00±0.00<br>Nf=2 | Mf=2<br>T1f=1002.34±4.57<br>af=24.50±4.50		N=2 | M=2<br>Nm=0 | Mm=0<br>T1m=0.00±0.00<br>am=0.00±0.00<br>Nf=2 | Mf=2<br>T1f=955.00±6.88<br>af=24.50±4.50		N=2 | M=2<br>Nm=0 | Mm=0<br>T1m=0.00±0.00<br>am=0.00±0.00<br>Nf=2 | Mf=2<br>T1f=1071.57±99.65<br>af=24.50±4.50						N=2 | M=4<br>Nm=2 | Mm=4<br>T1m=1253.86±19.29<br>am=42.00±11.00<br>Nf=0 | Mf=0<br>T1f=0.00±0.00<br>af=0.00±0.00
MOLLI<br>3(3)5 b									N=2 | M=2<br>Nm=0 | Mm=0<br>T1m=0.00±0.00<br>am=0.00±0.00<br>Nf=2 | Mf=2<br>T1f=1066.44±45.80<br>af=24.50±4.50		
MOLLI<br>4(1)3(1)2 b	N=2 | M=2<br>Nm=0 | Mm=0<br>T1m=0.00±0.00<br>am=0.00±0.00<br>Nf=2 | Mf=2<br>T1f=930.57±30.40<br>af=24.50±4.50		N=2 | M=4<br>Nm=0 | Mm=0<br>T1m=0.00±0.00<br>am=0.00±0.00<br>Nf=2 | Mf=4<br>T1f=910.63±25.09<br>af=24.50±4.50		N=1 | M=1<br>Nm=0 | Mm=0<br>T1m=0.00±0.00<br>am=0.00±0.00<br>Nf=1 | Mf=1<br>T1f=1163.15±0.00<br>af=29.00±0.00				N=2 | M=2<br>Nm=0 | Mm=0<br>T1m=0.00±0.00<br>am=0.00±0.00<br>Nf=2 | Mf=2<br>T1f=1137.49±62.97<br>af=24.50±4.50		
MOLLI<br>4(1)3(1)2 s	N=2 | M=2<br>Nm=0 | Mm=0<br>T1m=0.00±0.00<br>am=0.00±0.00<br>Nf=2 | Mf=2<br>T1f=967.59±4.28<br>af=24.50±4.50				N=2 | M=2<br>Nm=0 | Mm=0<br>T1m=0.00±0.00<br>am=0.00±0.00<br>Nf=2 | Mf=2<br>T1f=1204.71±8.58<br>af=24.50±4.50						
MOLLI<br>5(3)3 b	N=2 | M=2<br>Nm=0 | Mm=0<br>T1m=0.00±0.00<br>am=0.00±0.00<br>Nf=2 | Mf=2<br>T1f=1016.23±14.11<br>af=24.50±4.50	N=1 | M=1<br>Nm=0 | Mm=0<br>T1m=0.00±0.00<br>am=0.00±0.00<br>Nf=1 | Mf=1<br>T1f=970.15±0.00<br>af=23.00±0.00	N=7 | M=10<br>Nm=3 | Mm=3<br>T1m=982.06±20.32<br>am=40.33±14.08<br>Nf=4 | Mf=7<br>T1f=1000.00±26.39<br>af=39.00±17.65	N=10 | M=18<br>Nm=6 | Mm=7<br>T1m=974.08±13.63<br>am=37.00±14.01<br>Nf=4 | Mf=11<br>T1f=1009.06±19.58<br>af=33.50±12.84	N=2 | M=4<br>Nm=0 | Mm=0<br>T1m=0.00±0.00<br>am=0.00±0.00<br>Nf=2 | Mf=4<br>T1f=1234.62±16.57<br>af=24.50±4.50	N=3 | M=3<br>Nm=1 | Mm=1<br>T1m=1232.59±0.00<br>am=21.00±0.00<br>Nf=2 | Mf=2<br>T1f=1250.95±23.68<br>af=21.50±0.50		N=3 | M=13<br>Nm=1 | Mm=4<br>T1m=1193.51±8.64<br>am=30.00±0.00<br>Nf=2 | Mf=9<br>T1f=1211.32±9.44<br>af=21.00±1.00	N=2 | M=4<br>Nm=0 | Mm=0<br>T1m=0.00±0.00<br>am=0.00±0.00<br>Nf=2 | Mf=4<br>T1f=1258.41±43.10<br>af=24.50±4.50	N=3 | M=15<br>Nm=1 | Mm=4<br>T1m=1169.37±46.19<br>am=30.00±0.00<br>Nf=2 | Mf=11<br>T1f=1218.46±15.07<br>af=21.00±1.00	N=15 | M=27<br>Nm=7 | Mm=11<br>T1m=1158.42±29.43<br>am=47.14±17.45<br>Nf=8 | Mf=16<br>T1f=1151.09±37.02<br>af=44.62±17.63
MOLLI<br>5(3)3 s	N=2 | M=4<br>Nm=0 | Mm=0<br>T1m=0.00±0.00<br>am=0.00±0.00<br>Nf=2 | Mf=4<br>T1f=1021.46±8.42<br>af=24.50±4.50				N=2 | M=2<br>Nm=0 | Mm=0<br>T1m=0.00±0.00<br>am=0.00±0.00<br>Nf=2 | Mf=2<br>T1f=1271.09±17.32<br>af=24.50±4.50						N=5 | M=5<br>Nm=0 | Mm=0<br>T1m=0.00±0.00<br>am=0.00±0.00<br>Nf=5 | Mf=5<br>T1f=1091.83±24.00<br>af=31.00±2.97
SASHA	N=2 | M=2<br>Nm=0 | Mm=0<br>T1m=0.00±0.00<br>am=0.00±0.00<br>Nf=2 | Mf=2<br>T1f=1151.00±13.94<br>af=24.50±4.50		N=2 | M=2<br>Nm=0 | Mm=0<br>T1m=0.00±0.00<br>am=0.00±0.00<br>Nf=2 | Mf=2<br>T1f=1224.67±9.95<br>af=24.50±4.50	N=1 | M=3<br>Nm=0 | Mm=0<br>T1m=0.00±0.00<br>am=0.00±0.00<br>Nf=1 | Mf=3<br>T1f=1201.27±15.53<br>af=22.00±0.00	N=2 | M=2<br>Nm=0 | Mm=0<br>T1m=0.00±0.00<br>am=0.00±0.00<br>Nf=2 | Mf=2<br>T1f=952.68±5.23<br>af=24.50±4.50					N=3 | M=9<br>Nm=1 | Mm=3<br>T1m=1533.18±29.97<br>am=30.00±0.00<br>Nf=2 | Mf=6<br>T1f=1529.20±18.18<br>af=21.00±1.00	
SASHA GRE				N=3 | M=5<br>Nm=1 | Mm=2<br>T1m=1133.72±7.73<br>am=30.00±0.00<br>Nf=2 | Mf=3<br>T1f=1166.10±62.82<br>af=21.00±1.00							

S1.4: Patients with hypertrophic cardiomyopathy cohort (HCM) details with N=number of subjects, M=number of T1 maps, Nm=number of male subjects, Mm=number of male T1 maps, T1m=T1 time of male subjects [ms], am=age of male subjects [years], Nf=number of female subjects, Mf=number of female T1 maps, T1f=T1 time of female subjects [ms], af=age of female subjects [years], T1 values and age are given as mean±standard deviation, green denotes scanner-sequence-combinations represented in the training dataset

**N=112**<br>**M=121**	1.5T				3.0T						
	Philips<br>Achieva<br>[5.1.8.0]	Siemens<br>Avanto<br>[syngo MR B17]	Siemens<br>AvantoFit<br>[syngo MR D13B]	Siemens<br>AvantoFit<br>[syngo MR E11]	Philips<br>Ingenia<br>[5.1.8.2]	Philips<br>Ingenia<br>[5.4.0.0]	Philips<br>Ingenia<br>[5.4.0.1]	Siemens<br>PrismaFit<br>[syngo MR E11]	Siemens<br>Skyra<br>[syngo MR E11]	Siemens<br>SkyraFit<br>[syngo MR E11]	Siemens<br>Verio<br>[syngo MR B17]
MOLLI<br>3(3)3(3)5 b											
MOLLI<br>3(3)5 b											
MOLLI<br>4(1)3(1)2 b											
MOLLI<br>4(1)3(1)2 s											
MOLLI<br>5(3)3 b			N=47 | M=49<br>Nm=30 | Mm=31<br>T1m=990.68±43.03<br>am=46.27±10.42<br>Nf=17 | Mf=18<br>T1f=1023.02±35.43<br>af=52.18±8.26	N=28 | M=31<br>Nm=18 | Mm=20<br>T1m=1007.24±37.69<br>am=49.78±11.03<br>Nf=10 | Mf=11<br>T1f=1059.14±39.32<br>af=48.90±12.56							N=37 | M=41<br>Nm=23 | Mm=26<br>T1m=1192.52±51.19<br>am=52.43±11.78<br>Nf=14 | Mf=15<br>T1f=1223.51±47.96<br>af=53.21±8.45
MOLLI<br>5(3)3 s											
SASHA											
SASHA GRE											

S1.5: Patients with amyloidosis cohort (AMY) details with N=number of subjects, M=number of T1 maps, Nm=number of male subjects, Mm=number of male T1 maps, T1m=T1 time of male subjects [ms], am=age of male subjects [years], Nf=number of female subjects, Mf=number of female T1 maps, T1f=T1 time of female subjects [ms], af=age of female subjects [years], T1 values and age are given as mean±standard deviation, green denotes scanner-sequence-combinations represented in the training dataset

**N=24**<br>**M=24**	1.5T				3.0T						
	Philips<br>Achieva<br>[5.1.8.0]	Siemens<br>Avanto<br>[syngo MR B17]	Siemens<br>AvantoFit<br>[syngo MR D13B]	Siemens<br>AvantoFit<br>[syngo MR E11]	Philips<br>Ingenia<br>[5.1.8.2]	Philips<br>Ingenia<br>[5.4.0.0]	Philips<br>Ingenia<br>[5.4.0.1]	Siemens<br>PrismaFit<br>[syngo MR E11]	Siemens<br>Skyra<br>[syngo MR E11]	Siemens<br>SkyraFit<br>[syngo MR E11]	Siemens<br>Verio<br>[syngo MR B17]
MOLLI<br>3(3)3(3)5 b											
MOLLI<br>3(3)5 b											
MOLLI<br>4(1)3(1)2 b											
MOLLI<br>4(1)3(1)2 s											
MOLLI<br>5(3)3 b		N=1 | M=1<br>Nm=1 | Mm=1<br>T1m=1014.78±0.00<br>am=61.00±0.00<br>Nf=0 | Mf=0<br>T1f=0.00±0.00<br>af=0.00±0.00	N=13 | M=13<br>Nm=10 | Mm=10<br>T1m=1133.85±31.72<br>am=75.20±6.82<br>Nf=3 | Mf=3<br>T1f=1067.37±90.41<br>af=73.33±0.94	N=7 | M=7<br>Nm=7 | Mm=7<br>T1m=1108.40±42.22<br>am=73.71±10.31<br>Nf=0 | Mf=0<br>T1f=0.00±0.00<br>af=0.00±0.00							
MOLLI<br>5(3)3 s		N=2 | M=2<br>Nm=1 | Mm=1<br>T1m=1061.38±0.00<br>am=74.00±0.00<br>Nf=1 | Mf=1<br>T1f=1155.21±0.00<br>af=58.00±0.00									
SASHA											
SASHA GRE				N=1 | M=1<br>Nm=1 | Mm=1<br>T1m=1310.68±0.00<br>am=82.00±0.00<br>Nf=0 | Mf=0<br>T1f=0.00±0.00<br>af=0.00±0.00							
