# Supplementary material for: Post-hoc standardisation of parametric T1 maps in cardiovascular magnetic resonance imaging: a proof-of-concept
Source: eBioMedicine. 2024 Mar 14;102:105055. doi: 10.1016/j.ebiom.2024.105055 (PMC10951905; doi:10.1016/j.ebiom.2024.105055)
Supplement: S3 - MARISSA User Manual [file mmc3.pdf]

MARISSA

Magnetic Resonance Imaging Software for Standardization

User Manual

Version 1.0

12/02/2024

Authors:

Darian Viezzer[1,2,3,*]
Thomas Hadler[1,2,3]
Jan Gröschel[1,2,3]
Clemens Ammann[1,2,3]
Edyta Blaszczyk[1,2]
Christoph Kolbitsch[4]
Simone Hufnagel[4]
Riccardo Kranzusch-Groß[5]
Steffen Lange[6]
Jeanette Schulz-Menger[1,2,3,7]

[1] Charité – Universitätsmedizin Berlin, corporate member of Freie Universität Berlin and Humboldt-Universität zu Berlin, ECRC Experimental and Clinical Research Center, Lindenberger Weg 80, 13125 Berlin, Germany

[2] Working Group on Cardiovascular Magnetic Resonance, Experimental and Clinical Research Center, a joint cooperation between the Charité – Universitätsmedizin Berlin and the Max-Delbrück-Center for Molecular Medicine, Berlin, Germany

[3] DZHK (German Centre for Cardiovascular Research), partner site Berlin, Berlin, Germany

[4] Physikalisch-Technische Bundesanstalt Braunschweig and Berlin, Germany

[5] Universitätsklinikum Schleswig-Holstein, Klinik für Radiologie und Nuklearmedizin, Lübeck, Germany

[6] Hochschule Darmstadt (University of Applied Sciences), Faculty for Computer Sciences, Darmstadt, Germany

[7] Helios Hospital Berlin-Buch, Department of Cardiology and Nephrology, Berlin, Germany

* Corresponding author, E-Mail: darian-steven.viezzer@charite.de

Table of Contents

1 Introduction 3
2 License 3
3 Installation 4
4 Run 6
5 Workflow 6
5.1 Create a project 6
5.2 Setting up a standardization pipeline 6
5.3 Import data 7
5.4 Standardization pipeline training 7
5.5 Standardization pipeline application 7
6 Graphical User Interface 7
6.1 Start 8
6.2 Information 9
6.3 Project 10
6.4 Project Home 11
6.5 Setup 12
6.5.1 Parameters 12
6.5.2 Setups 14
6.6 Data 16
6.6.1 DICOM DATA 16
6.6.2 Segmentation 16
6.7 FAMD 20
6.8 Training 21
6.8.1 Setup 21
6.8.2 Data 22
6.8.3 Segmentation 23
6.8.4 Train 24
6.9 Train Plot 25
6.10 Standardize 26
6.10.1 External Data 27
6.10.2 Internal Data 28
7 Programming 29
7.1 homedirectory (Level 1) 29
7.1.1 appdata (Level 1.1) 29
7.1.2 marissa (Level 1.2) 30

1 Introduction

The Magnetic Resonance Imaging Software for Standardization (MARISSA) is an open-source software tool for the training, evaluation and application of post-hoc standardization pipelines on quantitative measurements in magnetic resonance imaging. A first proof-of-concept was performed on mid-ventricular native parametric T1 maps in cardiovascular magnetic resonance imaging, but it is intended to be applicable on other tissues and/or quantitative measurements.

While quantitative measurements are impacted by confounding parameters, MARISSA intends to transform those measurements into values of a reference confounding parameter environment. Therefore, the bias between the apparent measurement and the target transformation value is evaluated on a healthy volunteer population for different confounding parameters. This error is then subtractable, such that the quantitative values become comparable. However, the evaluation of the bias may be performed with different strategies. Therefore, MARISSA is a tool to run and evaluate those different standardization pipelines. Detailed information on the proof-of-concept is provided in the related publication by Viezzer et al.

The software tool was implemented in Python and can be installed on any common operating system (see section 3) while a graphical user interface (GUI) enables easy usability (see section 6).

2 License

MIT License

Copyright (c) 2023 Darian Steven Viezzer, Charité Universitätsmedizin Berlin

Permission is hereby granted, free of charge, to any person obtaining a copy of this software and associated documentation files (the "Software"), to deal in the Software without restriction, including without limitation the rights to use, copy, modify, merge, publish, distribute, sublicense, and/or sell copies of the Software, and to permit persons to whom the Software is furnished to do so, subject to the following conditions:

The above copyright notice and this permission notice shall be included in all copies or substantial portions of the Software.

THE SOFTWARE IS PROVIDED "AS IS", WITHOUT WARRANTY OF ANY KIND, EXPRESS OR IMPLIED, INCLUDING BUT NOT LIMITED TO THE WARRANTIES OF MERCHANTABILITY, FITNESS FOR A PARTICULAR PURPOSE AND NONINFRINGEMENT. IN NO EVENT SHALL THE AUTHORS OR COPYRIGHT HOLDERS BE LIABLE FOR ANY CLAIM, DAMAGES OR OTHER LIABILITY, WHETHER IN AN ACTION OF CONTRACT, TORT OR OTHERWISE, ARISING FROM, OUT OF OR IN CONNECTION WITH THE SOFTWARE OR THE USE OR OTHER DEALINGS IN THE SOFTWARE.

3 Installation

MARISSA was implemented with Python 3.8, hence we recommend to install the exact version number. If different, please ensure to have version 3.8 or higher. Python is available directly from the website of the Python Foundation:

https://www.python.org/downloads/

Besides python, necessary site-packages must be installed additionally. The package rasterio must be installed on Windows manually, Ubuntu and macOS user can skip this part. In Windows rasterio is installed as followed:

The rasterio installation is adapted from the instructions at https://sandbox.idre.ucla.edu/sandbox/tutorials/installing-gdal-for-windows:

- Install Visual Studio from https://visualstudio.microsoft.com/de/
- Install Visual Studio Build Tools 2019 and check during installation the workload C++ BuildTools by running in the command prompt: *winget install --id=Microsoft.VisualStudio.2019.BuildTools -e*
- Install VC-Redist from https://learn.microsoft.com/en-US/cpp/windows/latest-supported-vc-redist?view=msvc-170
- Install GDAL for windows from https://www.gisinternals.com/release.php
  - Choose the MSVC 2019 Compiler → x64 for 64bit Systems → release-1928-x64-gdal-3-5-3-mapserver-8-0-0 (could be different in future, just check that the year is according to the Visual Studio year and the number 1928 is equal or bigger than the MSVC version of your python)
  - Download and install the core msi: gdal-305-1928-x64-core.msi (could be different in the future)
  - Download and install the python libraries (be aware of your version, in this its 3.8): GDAL-3.5.3.win-amd64-py3.8.msi
- Add GDAL to system environment variables
  - Go to Start → search for variables → click on the variables button → go in the lower box (system variables)
  - Search for the Path entry → double click → Add new
  - Enter the Location of GDAL, i.e.: C:\Program Files\GDAL
  - Click ok
  - Under system variables click add new and insert the following two
    - Name: GDAL_DATA / Value: C:\Program Files\GDAL\gdal-data
    - Name: GDAL_DRIVER_PATH / Value: C:\Program Files\GDAL\gdalplugins
    - Ok and leave
- Overwrite Python GDAL with other version from https://www.lfd.uci.edu/~gohlke/pythonlibs/
  - Search for GDAL
  - Download the suitable GDAL wheel for your Python application, i.e. GDAL-3.4.3-cp38-cp38-win_amd64.whl
  - Install with command prompt:
    - *pip install <path to wheel>*
- Install Rasterio from https://www.lfd.uci.edu/~gohlke/pythonlibs/

    - Search for Rasterio
    - Download the suitable Rasterio wheel for your Python application, i.e. rasterio 1.2.10 cp38 cp38 win_amd64.whl
    - Install with command prompt:
        - *pip install <path to wheel>*
- Restart PC

For the usage of rasterio in python on Windows, even for other projects, please ensure to import gdal first, as for example:

from osgeo import gdal

import rasterio.features

The warnings saying “ERROR 1: Can’t load requested DLL” can be ignored.

After downloading the MARISSA source code from

https://github.com/DSV-CUB/marissa

run the install.bat (for Windows only). Alternatively start a command prompt, switch to the directory of the MARISSA software and run the command

pip install -r requirements.txt

This command will install all the necessary site packages:

```
absl-py==0.10.0
affine==2.3.0
astunparse==1.6.3
atomicwrites==1.4.0
attrs==21.4.0
backports.zoneinfo==0.2.1
branca==0.4.2
cachetools==4.1.1
certifi==2020.6.20
cffi==1.15.1
chardet==3.0.4
charset-normalizer==2.1.1
click==8.0.3
click-plugins==1.1.1
cligj==0.7.2
colorama==0.4.6
cycler==0.10.0
decorator==4.4.2
docopt==0.6.2
ERAlchemy==1.2.10
et-xmlfile==1.1.0
exceptiongroup==1.0.4
flatbuffers==2.0
fnv==0.2.0
folium==0.12.1.post1
fonttools==4.33.3
gast==0.3.3
geographiclib==1.52
geopy==2.2.0
google-auth==1.21.1
google-auth-oauthlib==0.4.1
google-pasta==0.2.0
googlemaps==4.6.0
graphviz==0.18.2
greenlet==1.1.3.post0
grpcio==1.32.0
h5py==2.10.0
idna==2.10
imageio==2.9.0
iniconfig==1.1.1
Jinja2==3.0.3
joblib==0.17.0
kiwisolver==1.2.0
libclang==12.0.0
light-famd==0.0.3
Markdown==3.2.2
MarkupSafe==2.1.1
matplotlib==3.5.2
mock==4.0.2
munch==2.5.0
munkres==1.1.4
networkx==2.5
numpy==1.23.5
oauthlib==3.1.0
opencv-contrib-python==4.6.0.66
opencv-contrib-python-headless==4.6.0.66
opencv-python==4.6.0.66
openpyxl==3.0.9
opt-einsum==3.3.0
packaging==21.3
pandas==1.5.2
patsy==0.5.2
Pillow==7.2.0
pipreqs==0.4.11
pluggy==1.0.0
polyline==1.4.0
prince==0.7.1
protobuf==3.13.0
py==1.11.0
py2opt==1.3.6
pyasn1==0.4.8
pyasn1-modules==0.2.8
pyclustering==0.10.1.2
pycparser==2.21
pydicom==2.2.2
pydot==1.4.2
pydotplus==2.0.2
pygraphviz==1.10
pyparsing==3.0.9
PyPDF4==1.27.0
pyproj==3.3.0
PyQt5==5.15.4
PyQt5-Qt5==5.15.2
PyQt5-sip==12.9.0
PyQt5-stubs==5.15.2.0
PyQt5Designer==5.14.1
pyqtgraph==0.12.3
PySide6==6.2.3
pytest==7.0.1
python-dateutil==2.8.1
python-magic-bin==0.4.14
pytz==2021.3
pytz-deprecation-shim==0.1.0.post0
PyWavelets==1.1.1
pywin32==302
qimage2ndarray==1.8.3
random2==1.0.1
requests==2.24.0
requests-oauthlib==1.3.0
rpy2==3.5.4
rsa==4.6
scikit-fuzzy==0.4.2
scikit-image==0.18.1
scikit-learn==0.23.2
scipy==1.10.1
seaborn==0.12.2
shapely==2.0.0
shiboken6==6.2.3
six==1.15.0
snuggs==1.4.7
SQLAlchemy==1.4.41
sqlalchemy-schemadisplay==1.3
statsmodels==0.13.0
tensorflow==2.7.0
termcolor==1.1.0
threadpoolctl==2.1.0
tifffile==2021.2.1
toml==2.0.1
typing==3.5.2.2
typing-extensions==4.0.1
tzdata==2022.2
tzlocal==4.2
urllib3==1.25.10
Werkzeug==1.0.1
wrapt==1.12.1
XlsxWriter==3.0.1
xlwings==0.25.0
xmltodict==0.13.0
yarg==0.1.9
yellowbrick==1.2.1
```

An optional site-package is pygraphviz to plot the database structure (see section 6.4), but it is not necessary for the overall functionality. If you want to use it, please install pygraphviz according to their homepage:

https://pygraphviz.github.io/documentation/stable/install.html

Additionally, MARISSA offers a factor analysis of mixed data (FAMD) via R (see section 6.7). This is an optional functionality and requires besides the rpy2 package the installation of R from

https://cran.r-project.org

and its packages NbClust, FactoMineR and factoextra. Information about installing packages in R are given on the R website.

4 Run

In Windows MARISSA can be started with the provided MARISSA.bat. Alternatively, start a command prompt, switch to the MARISSA folder and run the command:

python -m marissa.__init__

Both will start the GUI of MARISSA (see section 6). For batch processing, user with experience in Python programming can also run scripts. The scripts used in the proof-of-concept publication of this work are accessible in marissa\scripts. Further details on the implementation are given in the section 7.

5 Workflow

The workflow describes the typical order of using MARISSA to train a standardization pipeline and to apply this onto other data. Further information on the usage is given in the Graphical User Interface section (see section 6).

5.1 Create a project

The first step is to create a project. The user has to click on new in the start GUI (see section 6.1) and fill in the form to create a new project (see section 6.3). The project home is automatically started after creation (see section 6.4).

5.2 Setting up a standardization pipeline

From the project home a click on setup enables the definition of parameters that are accounted for standardization (see section 6.5.1) and setting up the pipeline settings (see section 6.5.2). While standard DICOM tags are already available, custom parameters based on DICOM tags are definable (see section 6.5.1.1). After definition of all considerable parameters, the standardization pipeline is set up (see section 6.5.2.1) by including additional information about regression model type, potential clustering strategy and the regression mode.

5.3 Import data

After definition of a standardization pipeline (see section 5.2), the data that is used for training of the standardization pipeline must be loaded into MARISSA via the data button in project home (see section 6.4). Additionally, test data is also already loadable. Beside the DICOM data, segmentation information must be included (see section 6.6). A meaningful description of the data is recommended in order to select the training data easily in the upcoming training step (see section 5.4). The same applies to the creator information for the segmentation data if more than one segmentation is loaded for a case.

5.4 Standardization pipeline training

After setting up the standardization pipeline (see section 5.2) and loading all necessary data into MARISSA (see section 5.3), the training button in project home (6.4) guides through the standardization pipeline training (see section 6.8). After selecting the standardization pipeline, the training data including the appropriate segmentations are chosen and finally the reference setup for each parameter is defined (see section 6.8). The training may take some time. The GUI is not usable during that and freezes. A notification will inform about the successful training of the standardization pipeline.

5.5 Standardization pipeline application

After training the standardization pipeline (see section 5.4), the standardization pipeline is applicable on new data. This new data can be either loaded into MARISSA (see section 5.3) or directly applied. Both is started from the standardize button in the project home (6.4).

6 Graphical User Interface

The GUI enables an easy interaction with the software. The following section shows the main GUI windows, its controls and describes how to use the software. In order to orientate with the programming section (see section 7) the individual GUIs are named in accordance with their respective ui and py filenames.

6.1 Start

After starting the software, the gui_start will show up as shown in Figure 1.

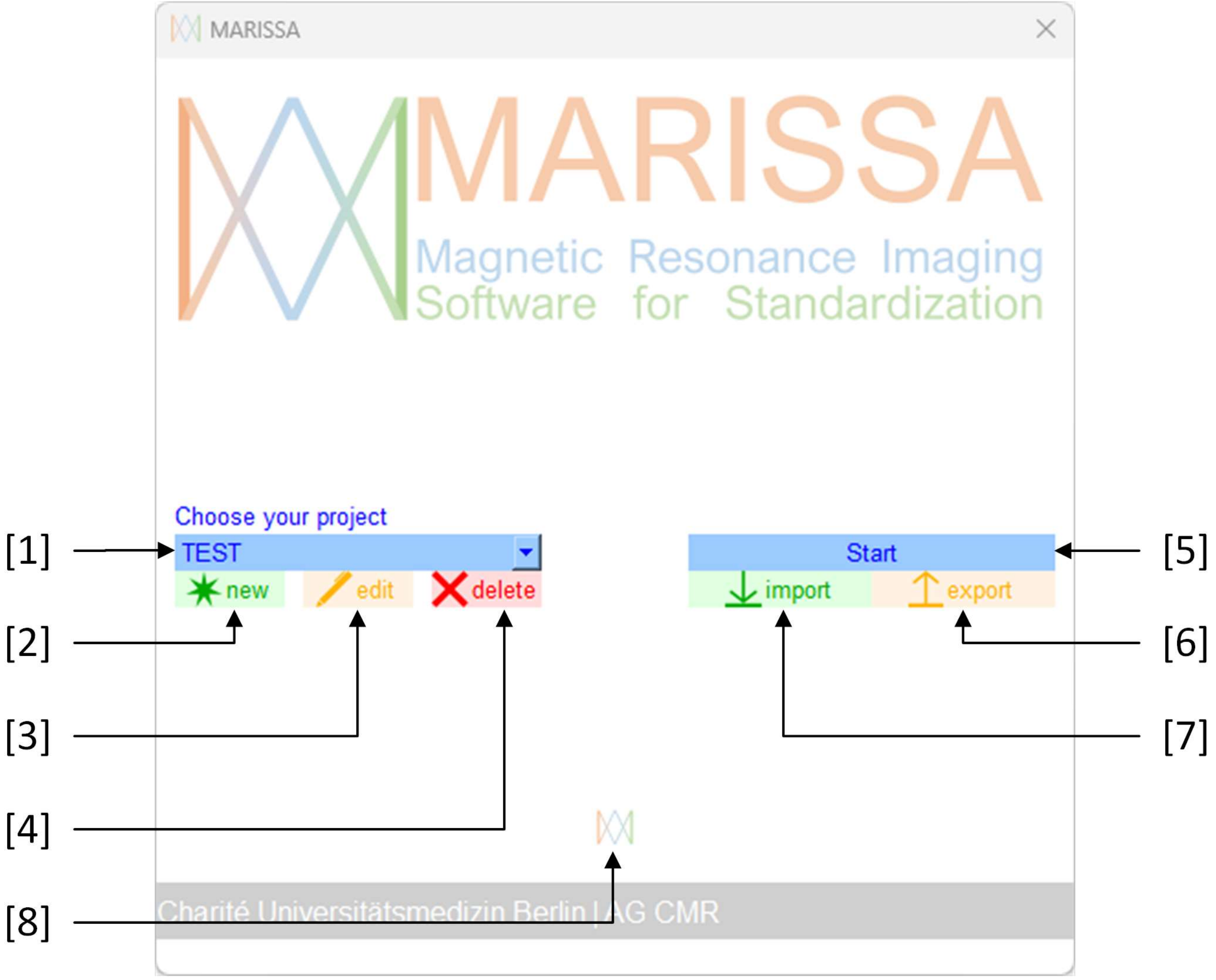

Figure 1: gui_start - [1] select project, [2] add new project, [3] edit project, [4] delete proect, [5] start project, [6] export project, [7] import roject and [8] information

Basically, MARISSA works in projects, such that different tissues, modalities, versions and so on can be separated. The field in [1] shows all existing projects. If none is given or a further should be started, then [2] adds a new one (see section 6.3). Editing a project via [3] will opens the same window. The deletion of a project via [4] is irreversible. To work on a project, the user need to chose one at [1] and click on start [5]. To share the project with others, [6] enables the export with or without data while [7] enables the import of a project. The exported file has the extension marissadb but represents a sqlite database and can be therefore accessed via any sqlite exploring tool. If an import project has the same name as an existing one, the existing one needs to be renamed via the edit function [2] otherwise the import fails. The information [8] shows the current information of MARISSA (see section 6.2)

6.2 Information

The dialog_info shows the information of the current MARISSA software as shown in Figure 2.

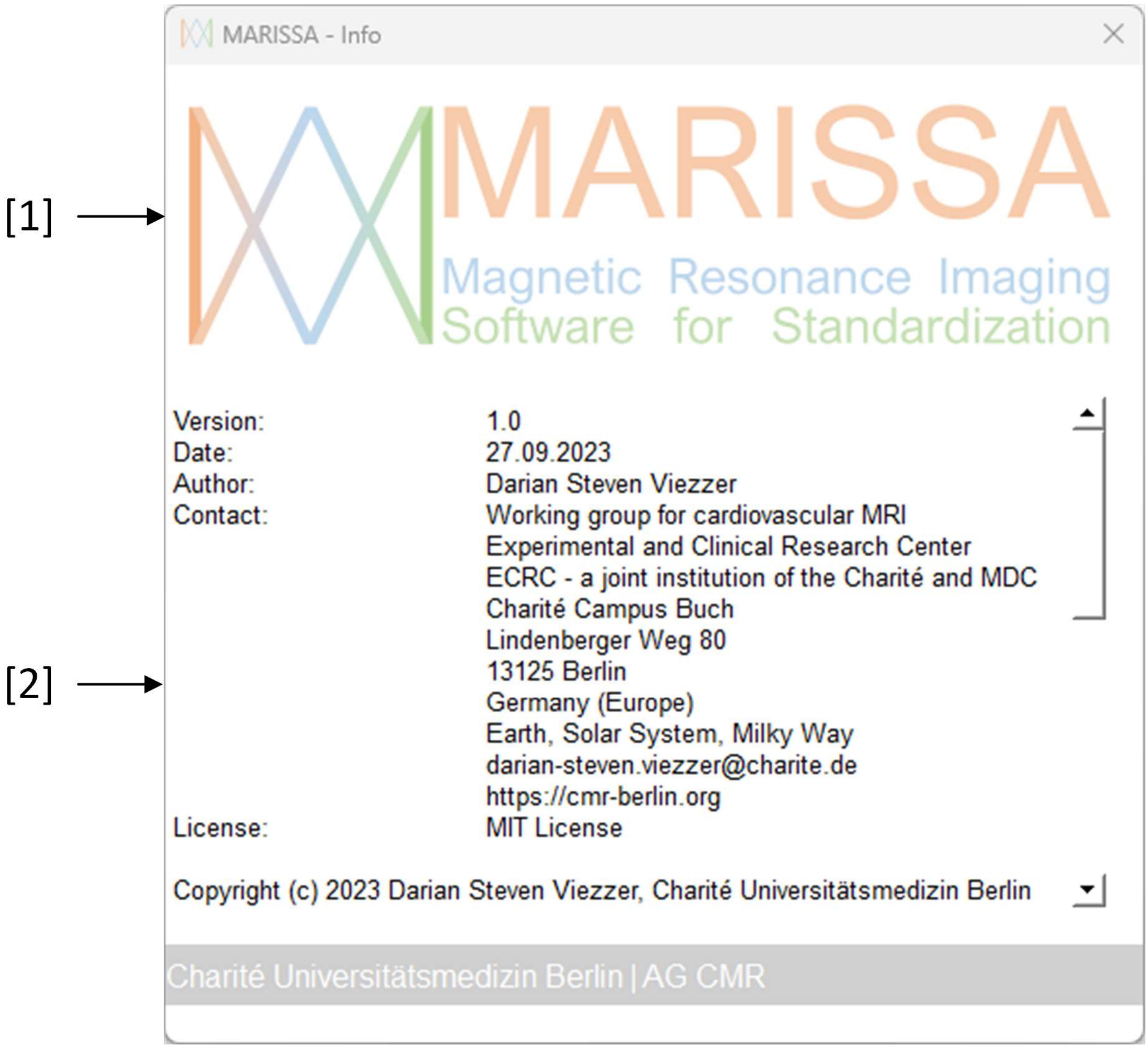

Figure 2: dialog_info – [1] MARISSA logo with link to the project homepage and [2] MARISSA information including the license with link to the working group website

A click on the logo [1] will open a browser to the project homepage

https://github.com/DSV-CUB/marissa

while a click in the information section [2] will open the webpage of the working group

https://cmr-berlin.org/en/en-home/

6.3 Project

Adding or editing a project will open the dialog_add_edit_project as shown in Figure 3.

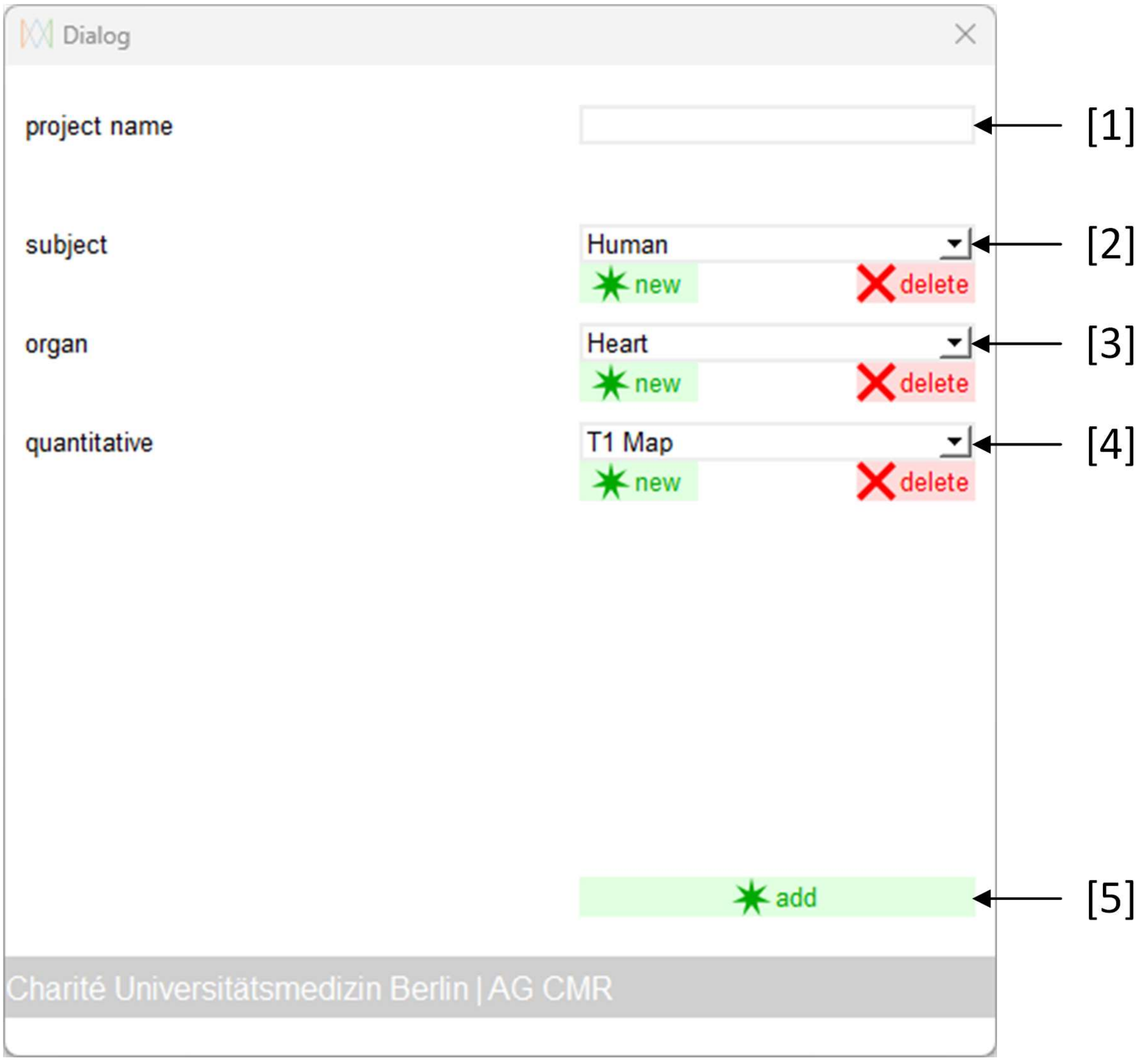

Figure 3: dialog_add_edit_project – [1] project name, [2] examination subject, [3] examination organ, [4] ecamination quantitative and [5] add espectively edit he project

When adding or editing a project (see section 6.1), a project name must be defined [1]. Every project must contain an information about the examined subject [2], organ [3] and quantitative [4]. Existing descriptions for these three fields can be added or deleted with the respective pushbuttons below. Although necessary, this information is not further used or of importance in the current version. The adding or editing [5] will open the project home (see section 6.4)

6.4 Project Home

The gui_project is the project home screen and appears whenever an existing project is started (see section 6.1), edited or a new one is created (see section 6.3). The following Figure 4 shows the appearing GUI window.

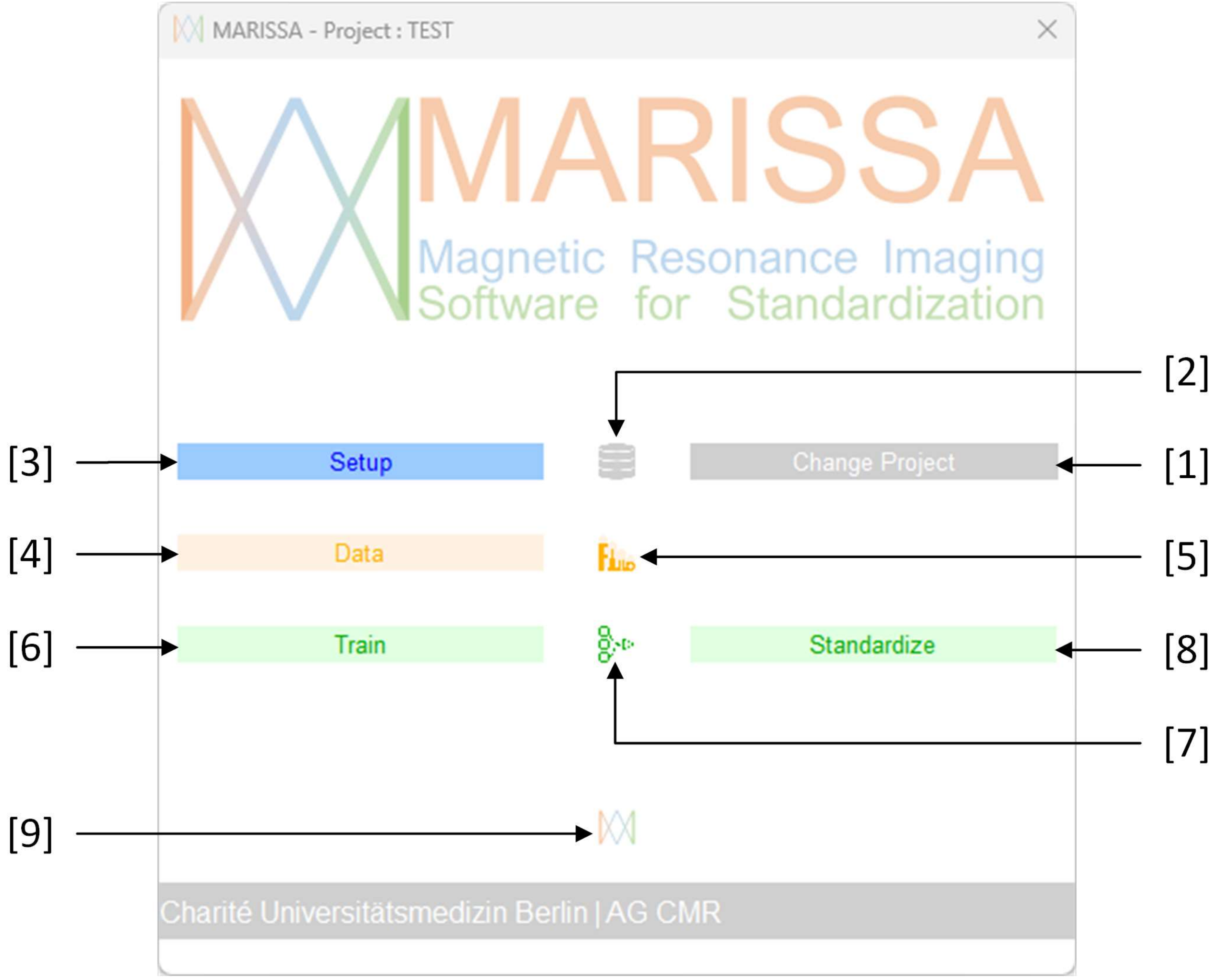

Figure 4: gui_project – [1] go to the start GUI, [2] print project database structure, [3] setup, [4] data, [5] factor analysis of mixed data, [6] train, [7] plot standardization training, [8] standardize and [9] information

To switch to another project the start window can be accessed via [1] (see section 6.1). Each project is a SQLite database in the background, the button [2] plots and saves the database structure. However, this requires pygraphviz to work (see section 3) and is only necessary if used for complex queries directly in the project database. However, the plot is limited such that it does not represent the full database structure. Consequently, this is still under construction.

The setup of the confounding parameters and the standardization pipeline is done via [3] (see section 6.5) while the DICOM data and corresponding segmentations can be loaded via [4] (see section 6.6). Existing segmented data can be analyzed via FAMD [5] (see 6.7) if the corresponding R package is installed (see section 3).

A standardization pipeline needs to be trained [6] (see section 6.8) while the regression plots can be accessed and exported [7] (see section 6.9). Application of the standardization procedure on internal or external data is possible [8] (see section 6.10). The information of MARISSA is also accessible from the gui_project [9] (see section 6.2).

6.5 Setup

In the gui_project_setup the confounding parameters as well as the setups of the standardization pipelines including the considered confounding parameters become defined.

6.5.1 Parameters

The primary view of the gui_project_setup is the parameters view as shown in Figure 5.

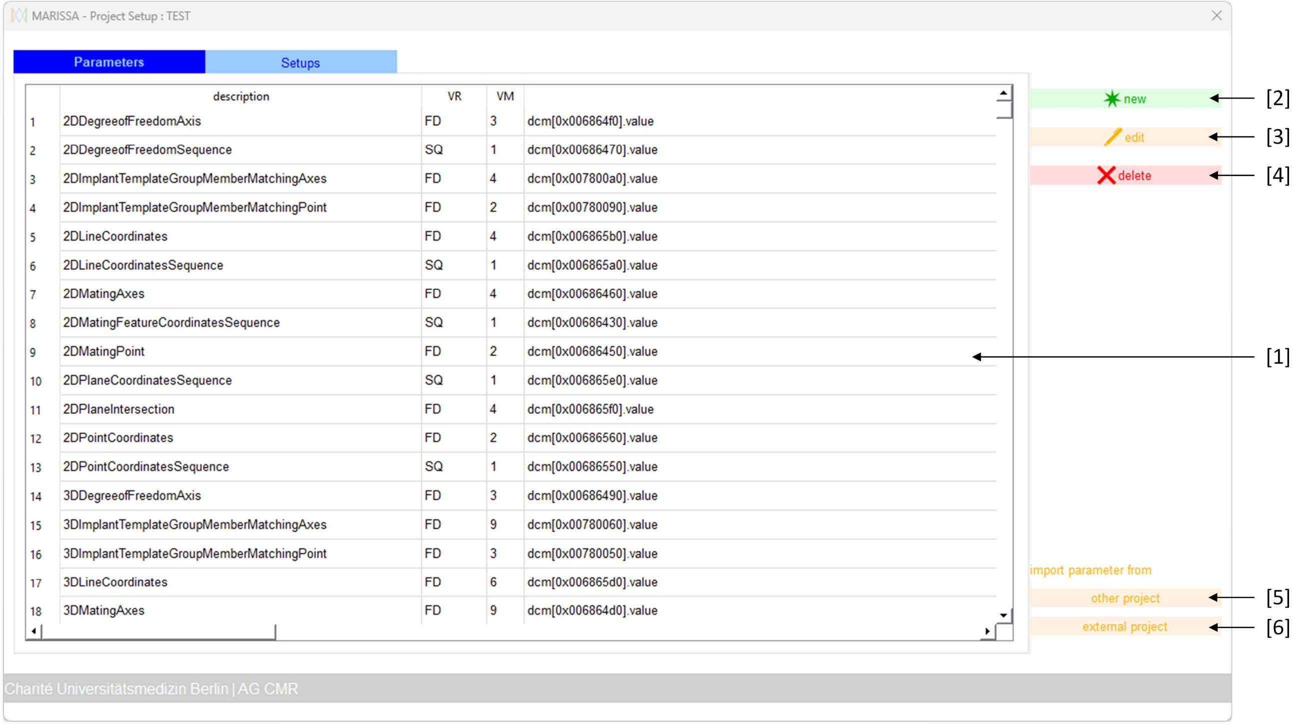

Figure 5: gui_project_setup - Parameters view – [1] parameters overview, [2] add parameter, [3] edit parameter, [4] delete parameter, [5] import parameters from another project and [6] import parameters from an external project

The overview of parameters [1] shows all parameters. A parameter must be extractable from a DICOM tag. MARISSA contains as a default all standard DICOM tags. A new parameter can be defined [2] or an existing can be edited (see section 6.5.1.1). Parameters can also be deleted [4]. Editing and deleting of parameters have no impact on already trained standardization pipelines as during training the information is copied separately. The import of parameters from other [5] or external [6] projects will import all not yet existing parameters.

6.5.1.1 Parameters Add/Edit

To add or edit a parameter a GUI window dialog_add_edit_parameter as in Figure 6 appears.

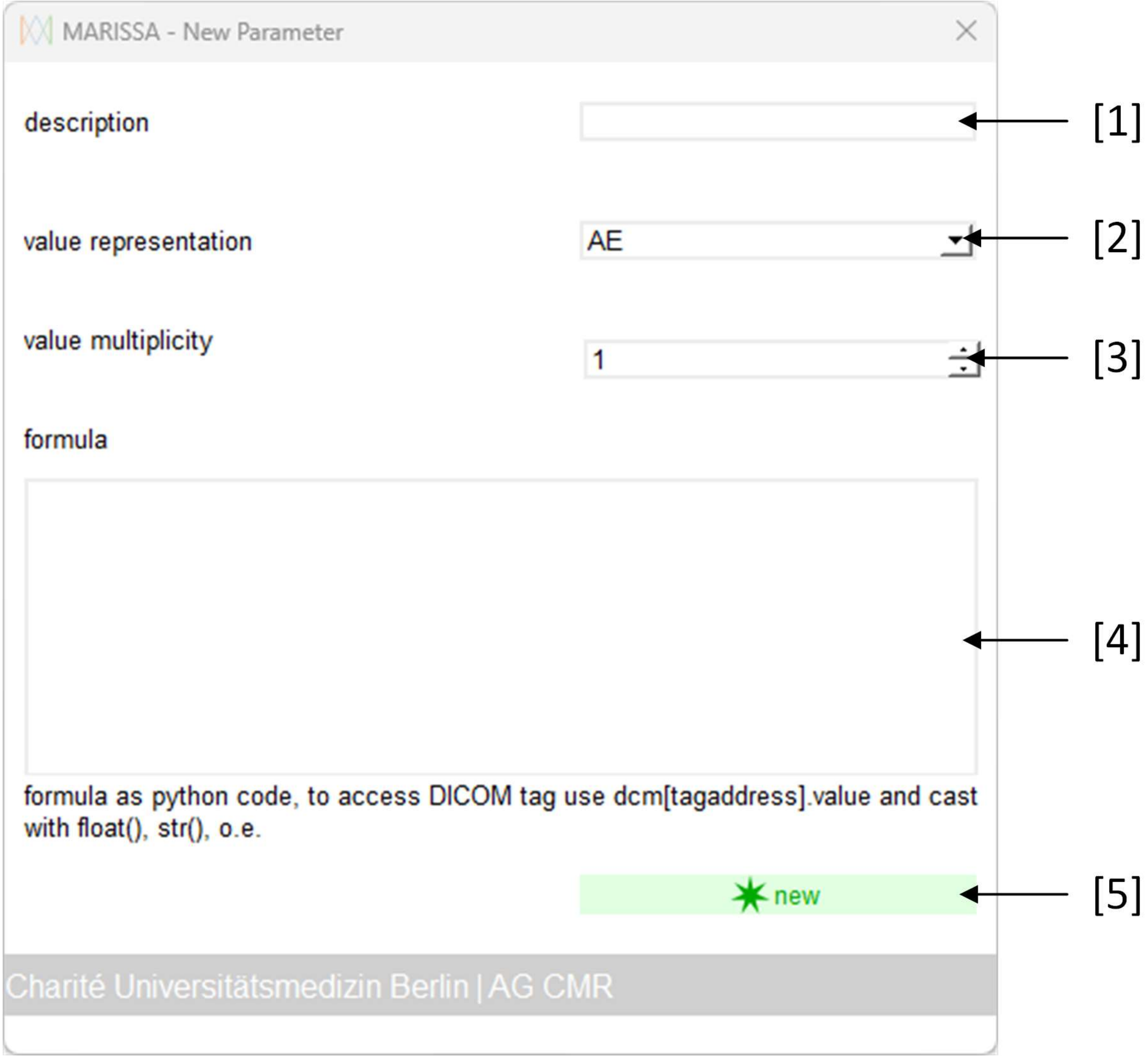

Figure 6: dialog_add_edit_parameter - [1] parameter description, [2] value representation according to the DICOM standard, [3] value multiplicity, [4] formula and [5] add or edit button

All fields are mandatory, the description [1] must be case-insensitive unique, all special characters are ignored. The value representation [3] defines whether the parameter is of categorical or numerical nature and how to read the DICOM tag data according to the DICOM standard. For more information please refer to

https://www.dicomstandard.org/current

The value multiplicity is necessary, if the DICOM tag contain multiple dimensions, as for example in the Pixel Spacing. MARISSA only works for fixed value multiplicities. When defining own parameters, the value multiplicity is usually one.

The formula [4] defines how to read the parameter. The most basic formular is to read directly a DICOM tag, i.e. to read the patient age attribute (it has the tag number 0x00101010) it can be accessed via:

dcm[0x00101010].value

The dcm is a placeholder for the DICOM file that was read with pydicom and the .value means that the value of this tag is needed otherwise the whole tag including value representation, etc. is contained. Self-defined parameters from one or multiple DICOM tags is possible but requires python programming skills, i.e. to access the first number in the patient age as an integer value, the formula would be:

int(str(dcm[0x00101010].value)[0])

There is currently no formula check, so a mistake in the formula definition is only visible during training. Finally, the parameter can be added or edited via [5]. The dialog window will close and the gui_project_setup will return in the parameters view.

6.5.2 Setups

After the definition of parameters (see section 6.5.1) the standardization pipeline setup must be defined. The Figure 7 shows the gui_project_setup in the Setups view that can be accessed via the blue tabs on the top.

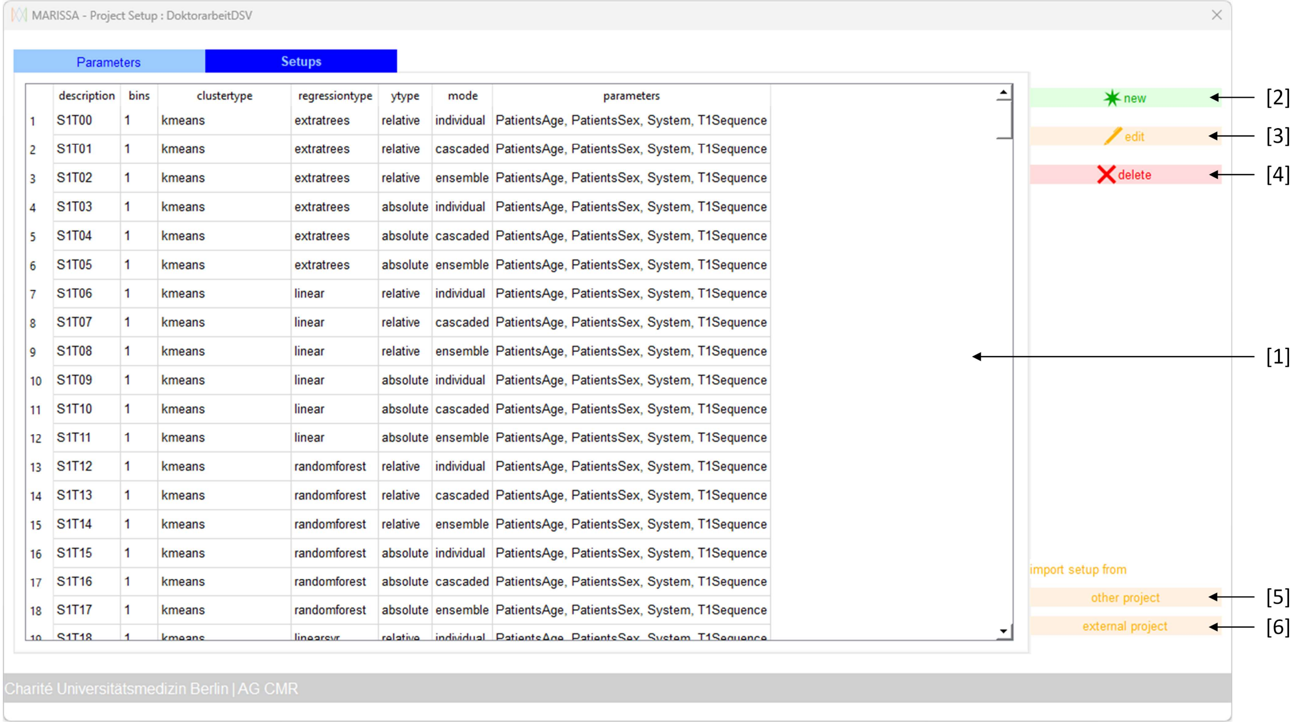

Figure 7: gui_project_setup - Setups view - [1] setups overview, [2] add setup, [3] edit setup, [4] delete setup, [5] import setup from another project and [6] import setup from an external project

The overview of setups [1] shows all parameters. A new setup can be defined [2] or an existing can be edited (see section6.5.2.1). Setups can also be deleted [4]. Editing and deleting of setups have no impact on already trained standardization pipelines as during training the information is copied separately. The import of setups from other [5] or external [6] projects will import all not yet existing setups.

6.5.2.1 Setups Add/Edit

To add or edit a parameter a GUI window dialog_add_edit_setup as in Figure 8 appears.

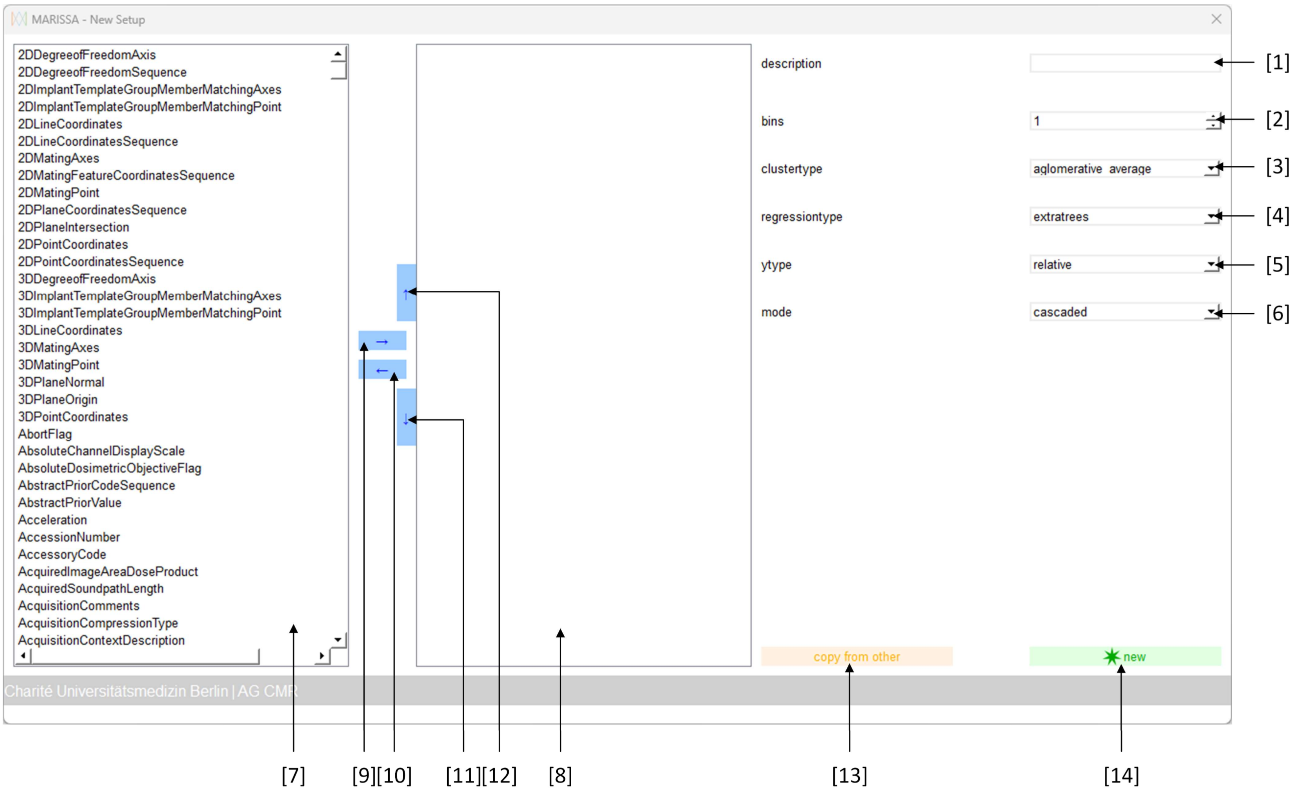

Figure 8: dialog_add_edit_setup - [1] setup description, [2] number of bins, [3] clustertype, [4] regressiontype, [5] ytype, [6] mode, [7] list of not considered parameters, [8] list of considered parameters, [9] add selected parameter to considered, [10] remove parameter from considered, [11] increase considered parameter position, [12] decrease considered parameter position, [13] copy setup settings from another setup and [14] add or edit the setup

All fields are mandatory, the description [1] must be case-insensitive unique, all special characters are ignored. The setup defines the strategy of the post-hoc standardization pipeline and is crucial for the performance. The setting of bins [2] larger than one requires the choice of the clustertype [3] as clustering method. This assures for different models for lower and larger values. The regressiontype [4] defines the regression model type that is used while the ytype [5] defines the model on either absolute or relative values. The mode [6] is used to work on either individual, cascaded or ensemble strategy. From the list of parameters [7] the confounding parameters [8] can be selected via [9] or vice versa via [10]. For the cascaded mode, the order of the confounding parameters is important, such that this can be set with [11] and [12]. The copy [13] function allows to adopt the settings of another existing pipeline. Finally, the setup can be added or edited via [14].

6.6 Data

In the gui_project_data the DICOM data as well as the segmentation become imported into MARISSA.

6.6.1 DICOM DATA

The primary view of the gui_project_data is the parameters view as shown in Figure 9.

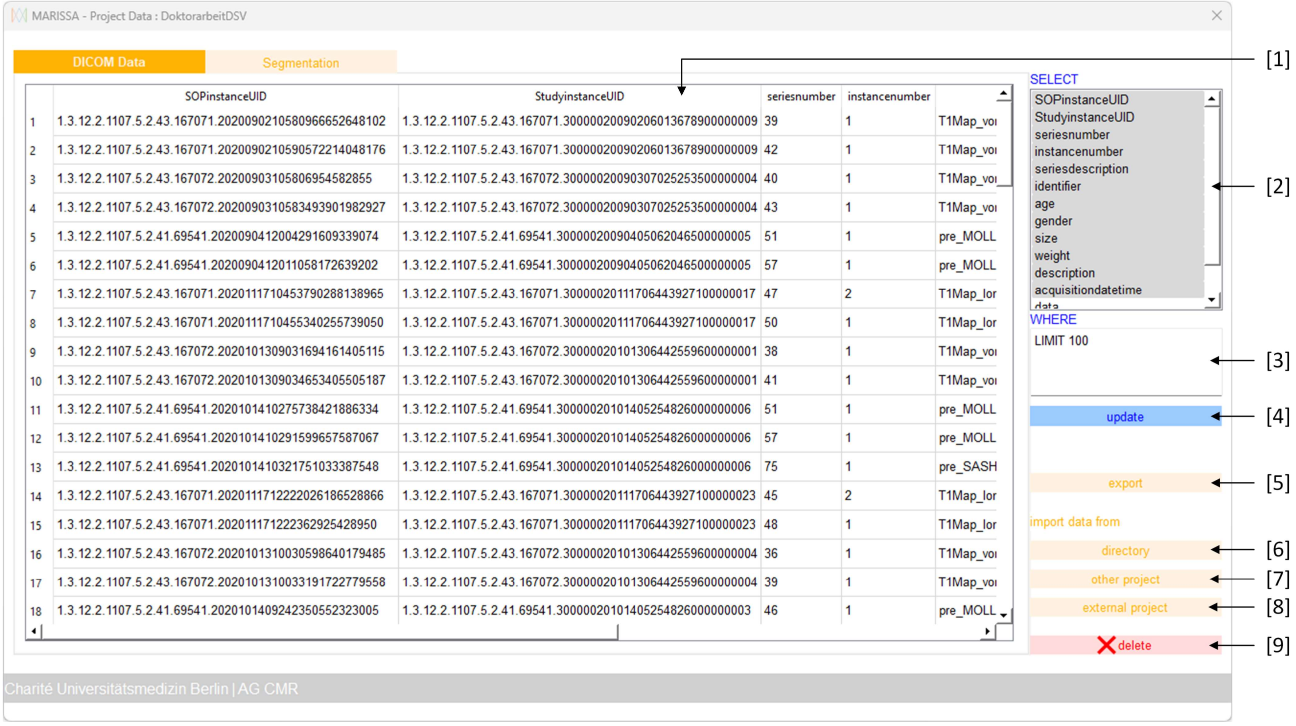

Figure 9: gui_project_data – DICOM data view - [1] DICOM overview, [2] list columns selection, [3] filter data, [4] update the overview according to the filter, [5] export data, [6] import data from a directory, [7] import data from another project, [8] import data from an external project and [9] delete data that is shown in the overview

The overview of data [1] shows all selected data. By default it shows the first 100 data in the database. The column selection [2] enables to select the columns of interest for displaying. A filtering via an SQL WHERE clause [3] will be applied with the click on the update button [4]. A double click on a dataset in the overview [1] opens a plot of the data. The data shown in the overview [1] can be exported as DICOM files [5]. An import is either done from directory [6], another project [7] or an external project [8]. The deletion [9] is irreversible and applies on all data in the overview [1]. To delete single dataset, the usage of filtering [3] is necessary.

6.6.2 Segmentation

After the import of data (see section 6.6.1) the corresponding segmentation must be defined. The shows the gui_project_data in the Segmentation view that can be accessed via the orange tabs on the top.

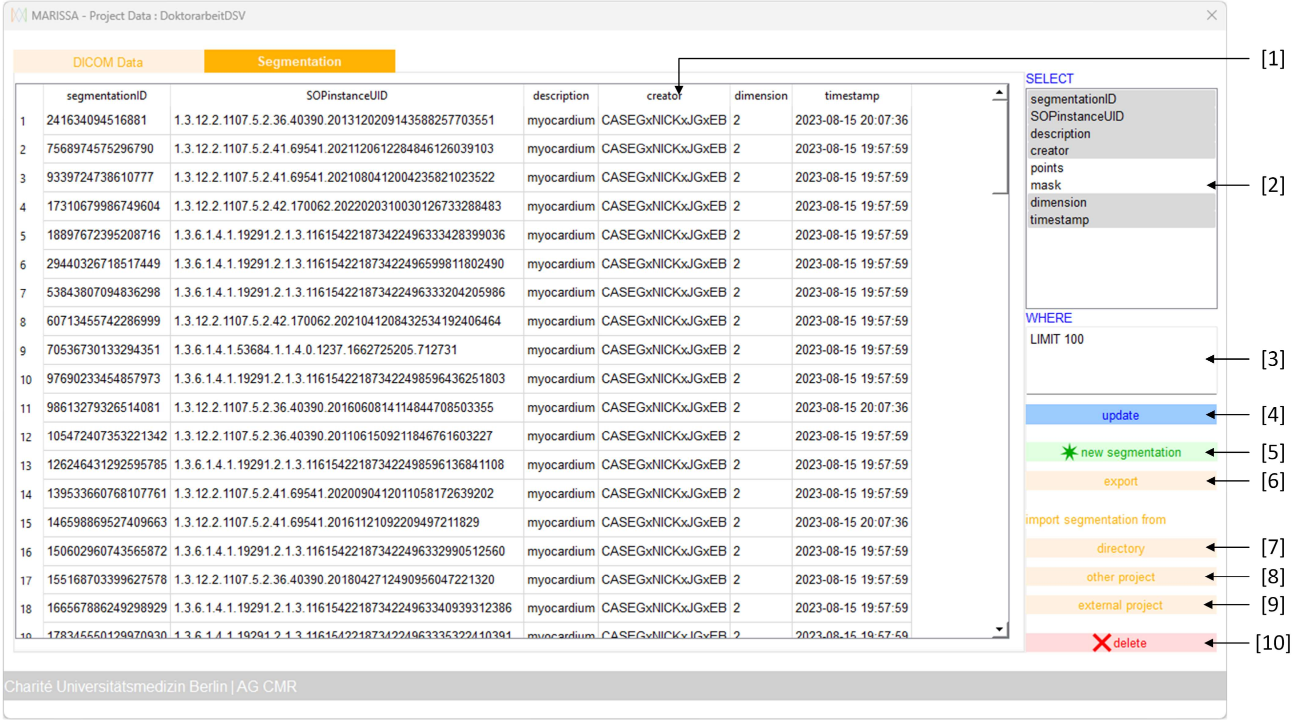

Figure 10: gui_project_data – Segmentation view - [1] segmentation data overview, [2] list columns selection, [3] filter data, [4] update the overview according to the filter, [5] create new segmentation rule, [6] export segmentation, [7] import segmentations from a directory, [8] import segmentations from another project, [9] import segmentations from an external project and [10] delete segmentations that is shown in the overview

The overview of segmentations [1] shows all selected segmentations. By default it shows the first 100 segmentations in the database. The column selection [2] enables to select the columns of interest for displaying. A filtering via an SQL WHERE clause [3] will be applied with the click on the update button [4]. A double click on a segmentation in the overview [1] opens a plot of the corresponding data and the contour. Multiple segmentations can be combined with the new segmentation button [5] that opens the segmentation rule (see section 6.6.2.2). The segmentation shown in the overview [1] can be exported as numpy or pickle files [6]. An import is either done from directory [7] (see section 6.6.2.1), another project [8] or an external project [9]. The deletion [10] is irreversible and applies on all segmentations in the overview [1]. To delete single segmentations, the usage of filtering [3] is necessary.

6.6.2.1 Segmentation Import

The import of segmentations require either numpy or pickle file formats. Figure 11 shows the import window.

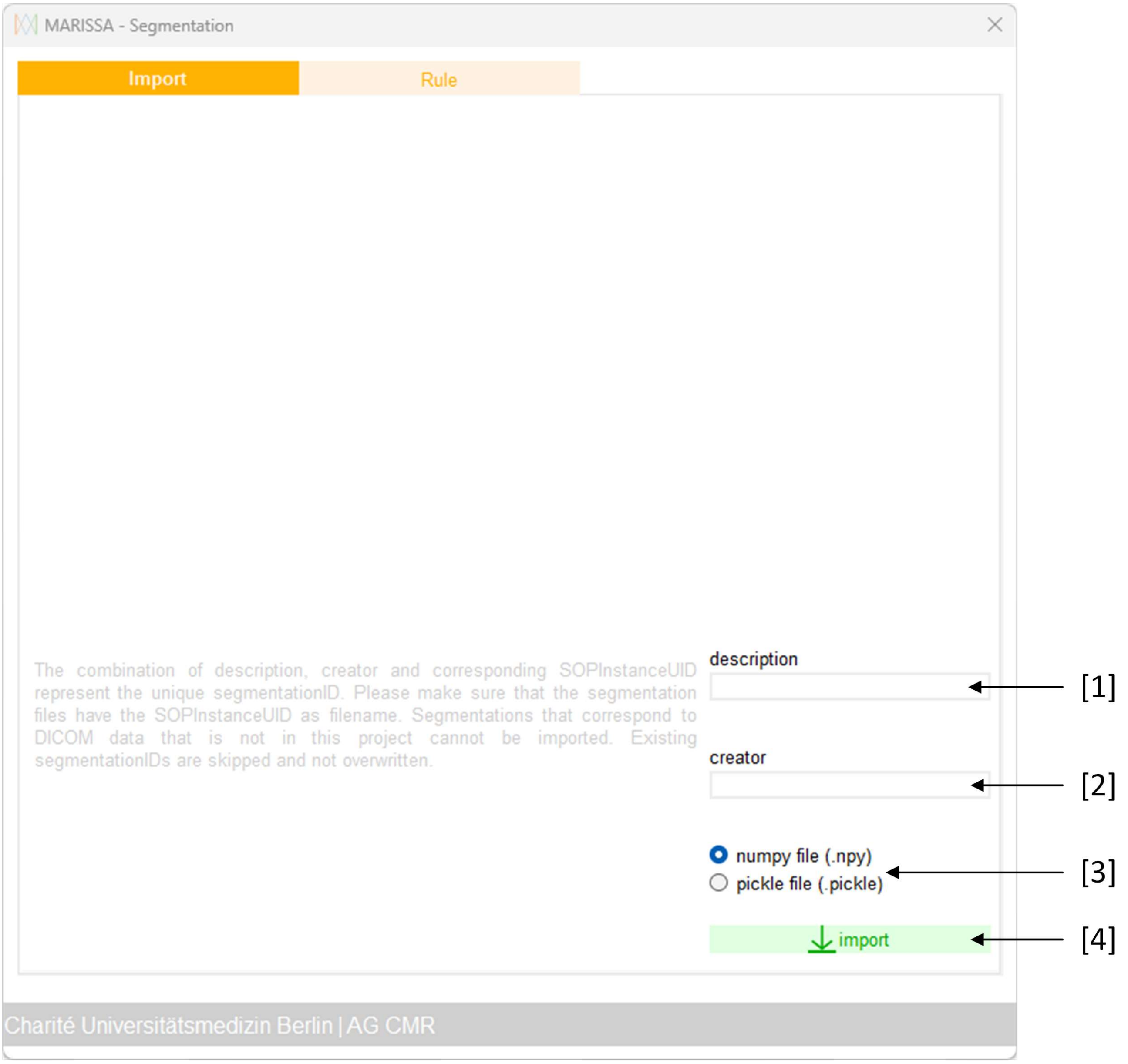

Figure 11: dialog_add_edit_segmentation – Import view: [1] description, [2] creator of the segmentation, [3] file format and [4] import segmentation

The import of segmentations requires a description [1] and information about the creator [2]. The choice can be arbitrary although special characters are deleted. The file type [3] defines the way to import segmentations. Numpy files are pickled binary numpy arrays of the same size as the corresponding DICOM images. On the contrary pickle files are dictionaries with a *lv_myo* dict attribute that contains the *cont* and *imageSize* attributes. The *cont* represents shapely polygons of the segmentation and *imageSize* the size of the corresponding DICOM data. Although historically available, we recommend to use the numpy file import. In any way,

the filename must represent the SOPinstanceUID of the DICOM data that belongs to the segmentation as otherwise the match cannot be made. A click on import [4] asks the user for the directory in which the segmentations are. Please note, that the algorithm runs also through all sub-directories.

6.6.2.2 Segmentation Rule

As multiple segmentation methods may exist for the same dataset, MARISSA enables the setup of rules to create novel segmentations.

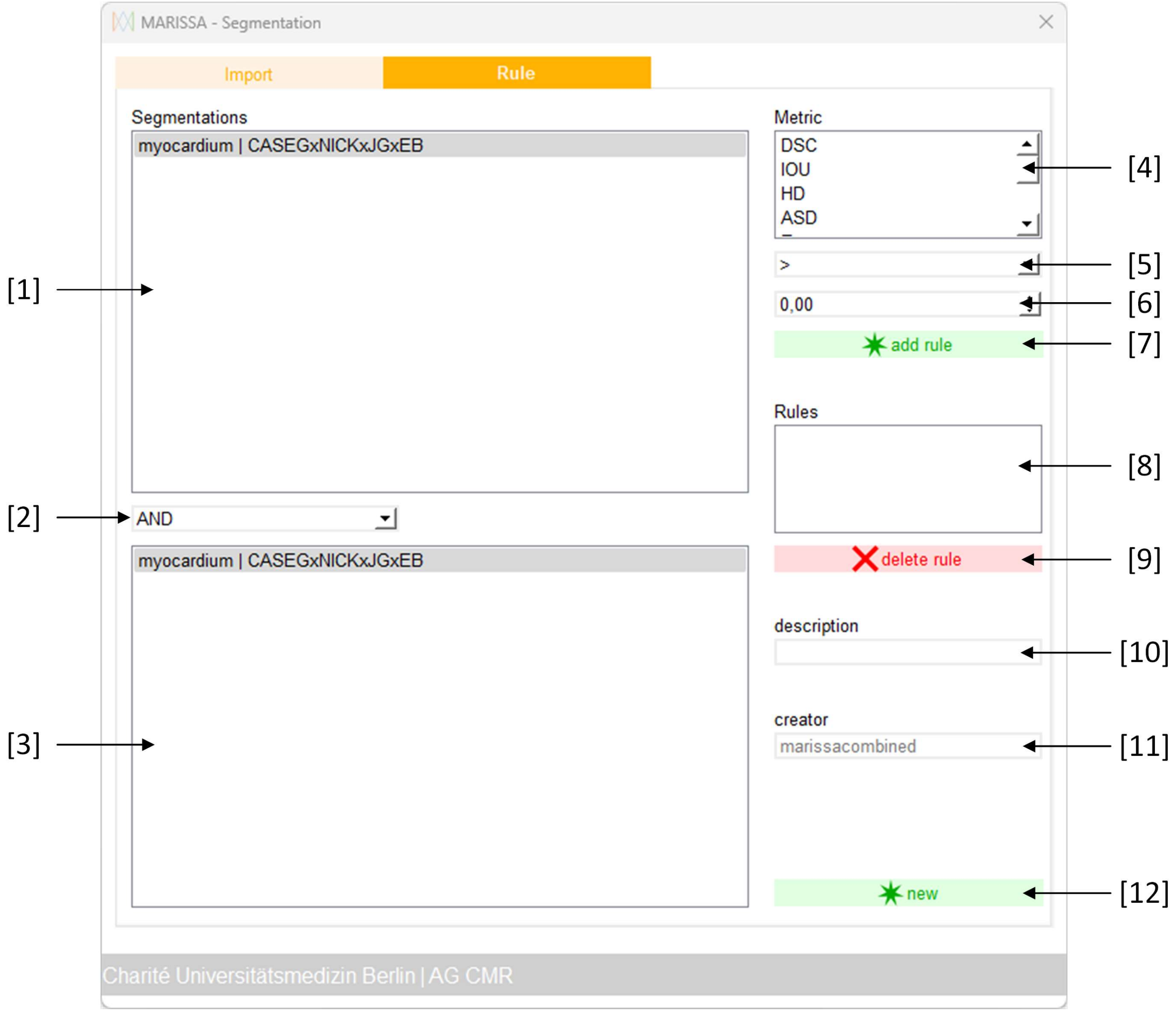

Figure 12: dialog_add_edit_segmentation – Rule view: [1] segmentation 1, [2] logical connector, [3] segmentation 2, [4] rule metric, [5] rule metric operator, [6] rule metric threshold, [7] adding the rule metric, [8] list of rules, [9] deleting rule metric, [10] description, [11] creator and [12] save button

Two segmentations, denoted by [1] and [2], can be logically connected [3] to a new segmentation. The setting of a rule applies to only a part of the data that fulfill the metric rules. The metric [4] is selectable with an operator [5] and a threshold [6] by adding as a rule. Please note that the metrics have different units. Only if the two segmentations are valid for all rules

[8], then the connected segmentation is saved. A rule can be deleted [9]. The new segmentation requires a description [10] while the creator is pre-defined. A click on the new button [12] will evaluate all new segmentations.

6.7 FAMD

If R and the corresponding packages (see section 3) is installed, then a Factor Analysis on Mixed Data (FAMD) can be performed. The FAMD GUI is shown in

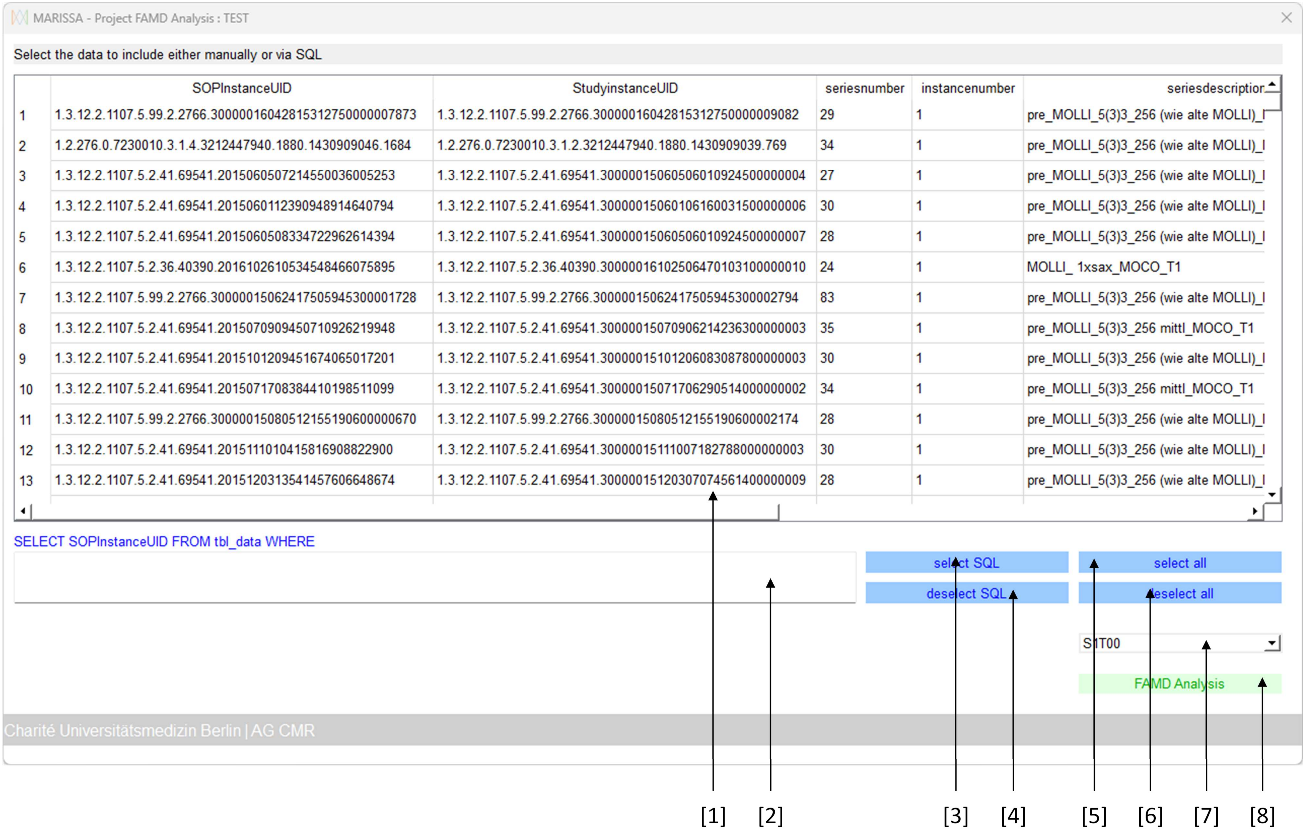

Figure 13: gui-project_famd: [1] list of data, [2] filter field, [3] select all data according to the filter, [4] deselect data according to the filter, [5] select all data, [6] deselect all data, [7] standardization pipeline and [8] run the FAMD analysis

The FAMD analysis the potential impact weight of confounding parameters. The analysis is applied on the selected data in the overview [1]. Selection can be either done manually by clicking or via the SQL filtering [2] and the select [3] or deselect [4] buttons. All data at once are directly selectable [5] or de-selectable [6]. The choice of the standardization pipeline [7] is necessary to select the confounding parameters. The run [8] of the FAMD analysis exports the resulting plots of representation of Individuals, Relationship Square, Correlation Circle and Representation of categories.

6.8 Training

In the gui_project_training the training of the standardization pipelines is organized and performed. During training, the software currently freezes and cannot be used for that moment.

6.8.1 Setup

MARISSA guides through the necessary steps for the training of a standardization pipeline starting with the setup choice as shown in Figure 14.

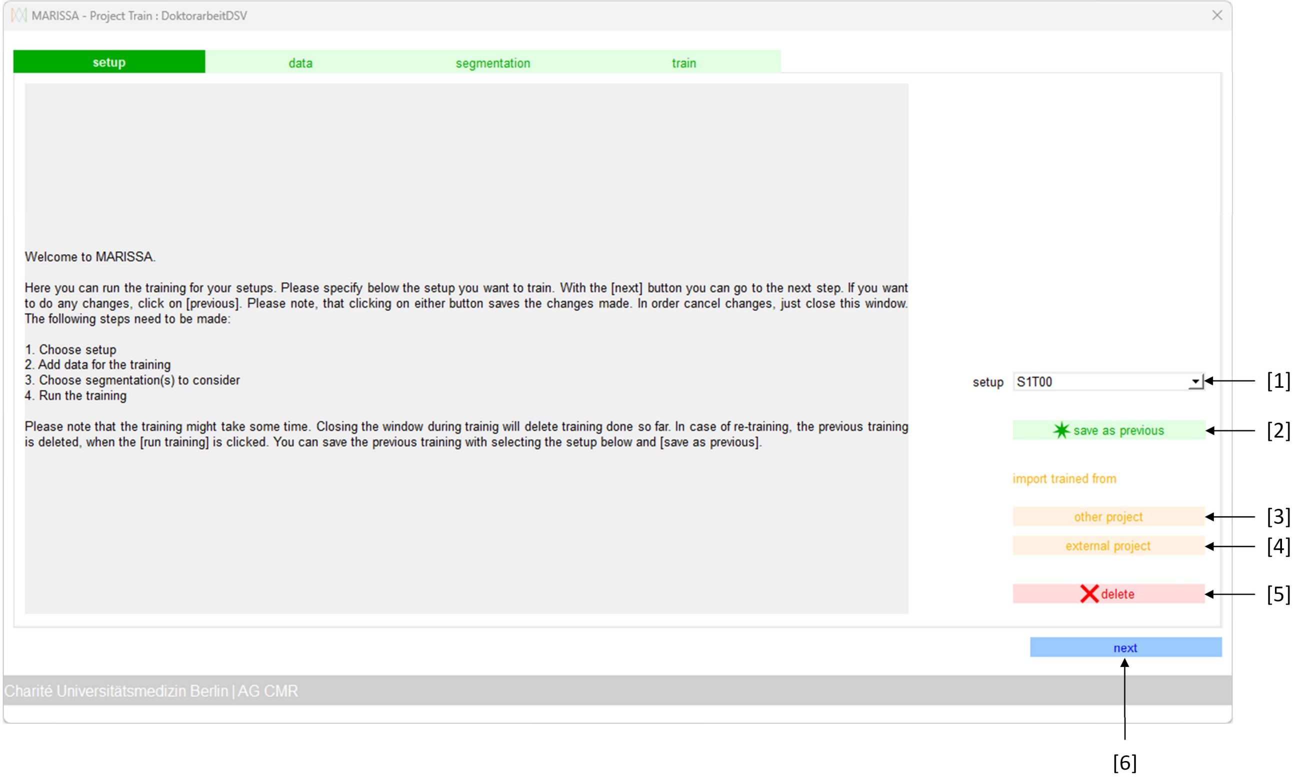

Figure 14: gui_project_training – setup view: [1] standardization pipeline setup, [2] save a copy, [3] import standardization pipeline from another project, [5] import standardization pipeline from an external project, [5] delete trained standardization pipeline and [6] go to the next step

The choice of the standardization pipeline [1] is the beginning. If it was already trained and should be backed up, then the save as a previous [2] will do that. Standardization pipelines can also be imported from another project [3] or an external project [4]. The deletion of a trained standardization pipeline is also available [5]. Clicking on the next [6] button will go to the standardization pipeline training data match (see section 6.8.2).

6.8.2 Data

After choice of the standardization pipeline, the training data can be selected as shown in Figure 15.

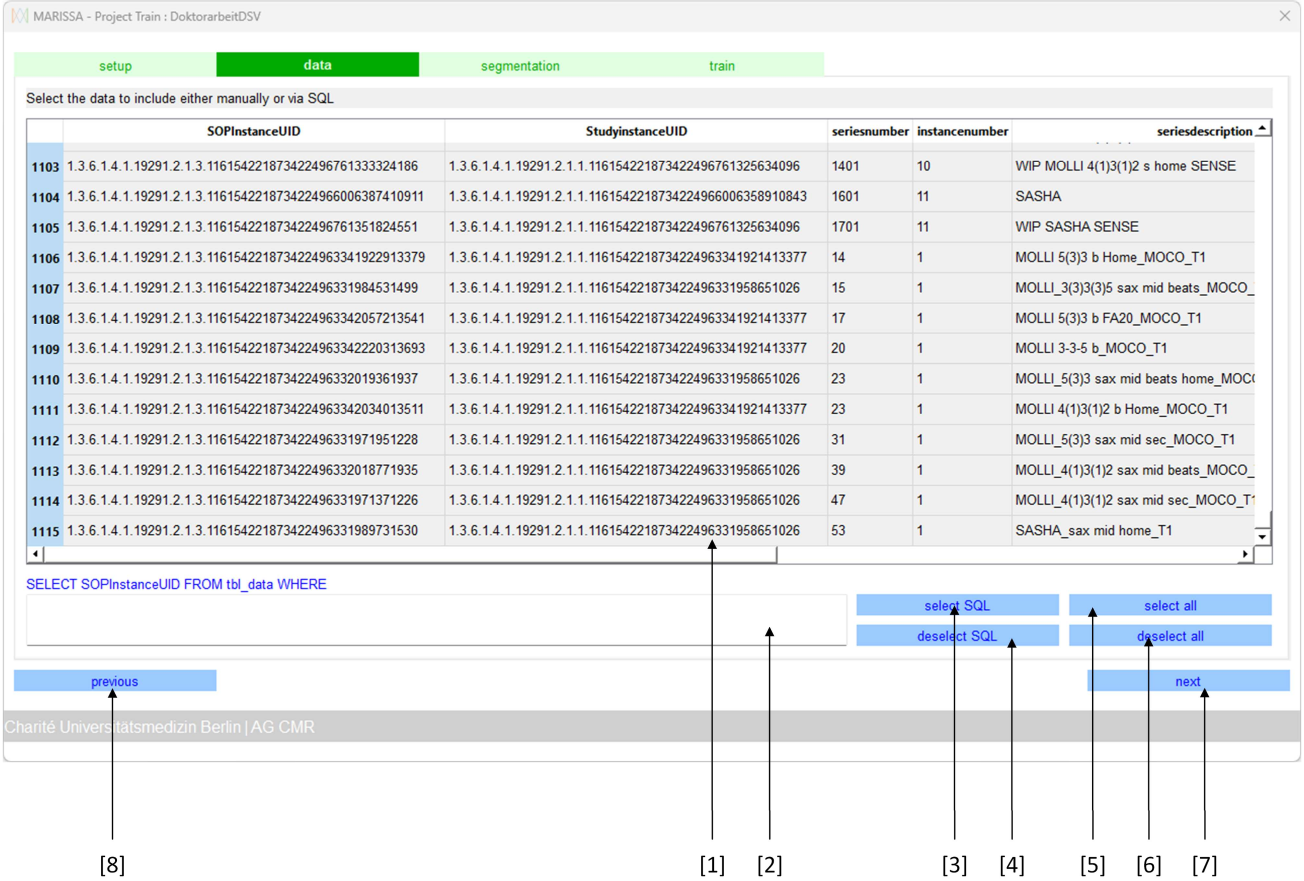

Figure 15: gui_project_training – data view: [1] DICOM data overview, [2] SQL filter, [3] select filtered data, [4] deselect filtered data, [5] select all data, [6] deselect all data, [7] go to the next step and [8] go to the previous step

The training data for the standardization pipeline can be selected manually from the overview [1] or by applying a SQL filter [2] to select [3] or deselect [4] the queried data. Selecting all [5] and deselecting all [6] is also possible. Clicking on the next [7] button will go to the standardization pipeline training data segmentation match (see section 6.8.3), while clicking on previous will return to the setup view (6.8.1).

6.8.3 Segmentation

After choice of the standardization pipeline training data, the corresponding segmentations are chosen as shown in Figure 16.

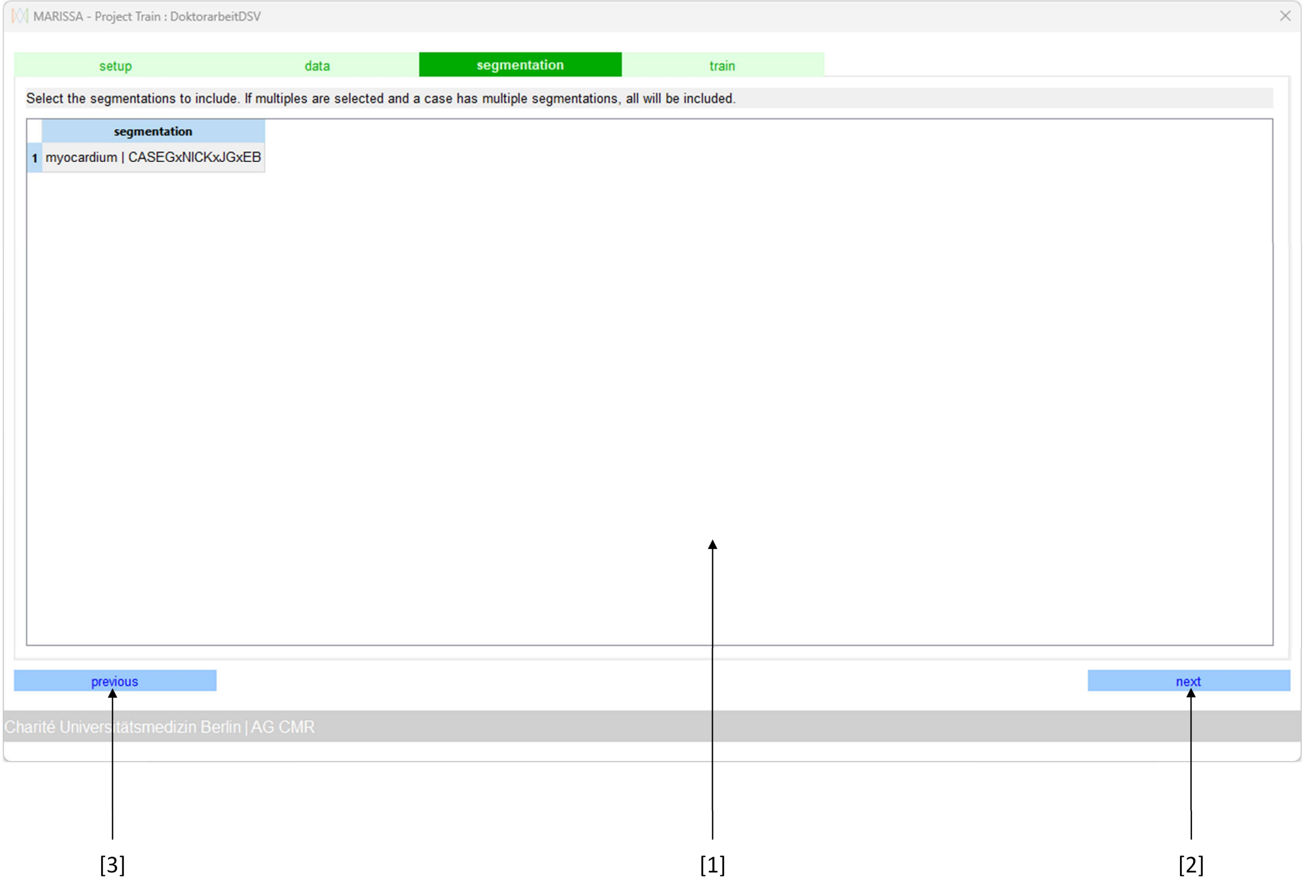

Figure 16: gui_project_training – segmentation view: [1] segmentation overview, [2] go to the next step and [3] go to the previous step

Choosing the segmentation is based on the combination of description and creator (see section 6.6.2). If multiple segmentations are chosen and a dataset has multiple segmentations, then all of them are used, so the dataset is multiple times in the training used with different segmentations. If, on the contrary, a chosen dataset (see section 6.8.2) has no segmentation, then the dataset is omitted. Clicking on the next [2] button will go to the standardization pipeline training overview (see section 6.8.4), while the next button [3] goes back to the training data selection (see section 6.8.2).

6.8.4 Train

After initializing the standardization pipeline training with the training data and segmentations, an overview is shown as in

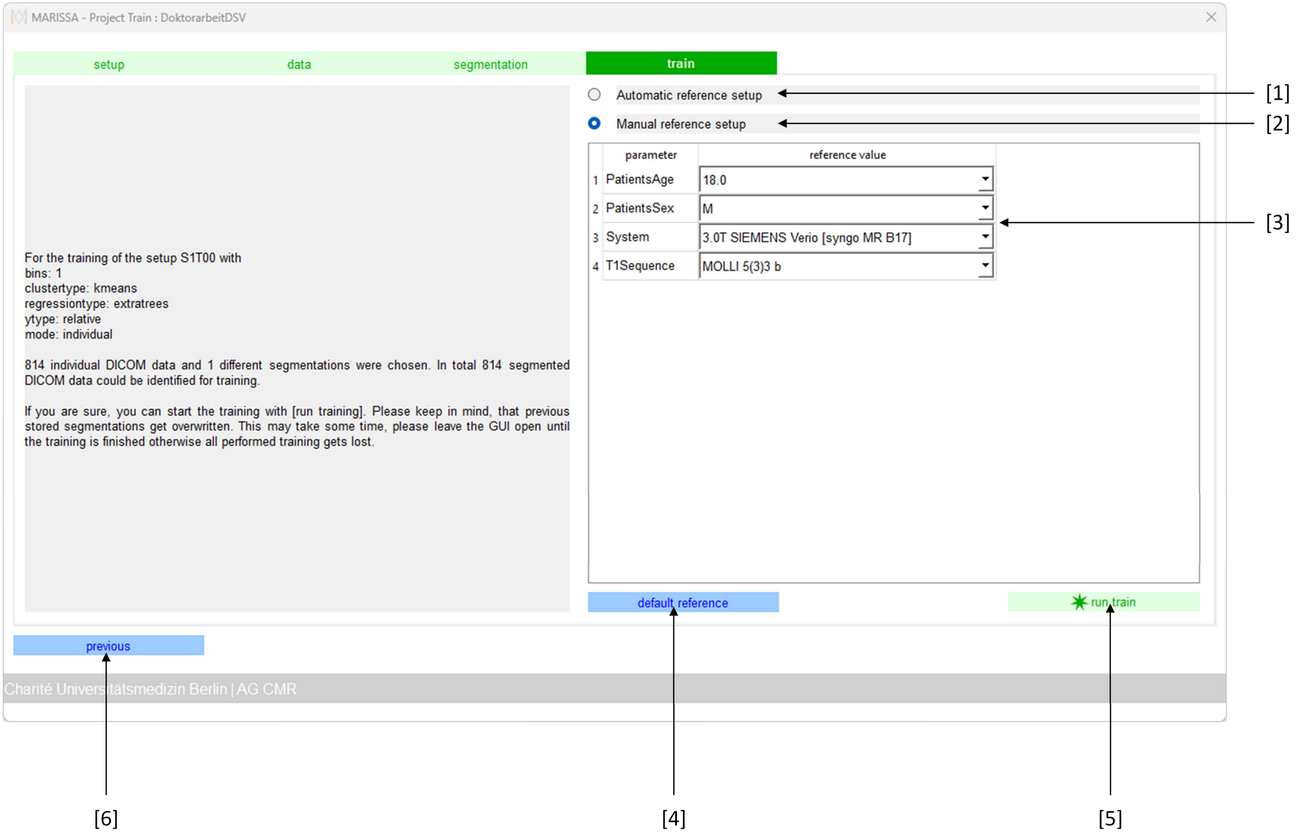

Figure 17: gui_project_training – train view: [1] automatical reference setup, [2] manual reference setup, [3] manual reference setup list, [4] default reference setup, [5] start training and [6] go to the previous step

On the left-hand site all information about the standardization pipeline including the number of considered training data is listed. Regarding the reference confounding parameter environment, either an automatic [1] or a manual [2] mode is possible. The automatic mode just takes the setup that is most occurring across the training dataset. In the manual, each confounding parameter can be adjusted [3]. However, the combination must be a valid combination that exists across the training data. Consequently, the assortment for the second parameter depends on the value of the first, the third parameter on the first and second and so on. A click on default reference [4] will fill in the manual mode [3] the default values / automatic reference setup. A click on train [5] starts the training. During that time, MARISSA GUI freezes and is not usable. The user gets informed, when the training was performed and the GUI switches automatically back to the setup view (see section 6.8.1). A click on previous [6] goes back to the training data segmentation selection (see section 6.8.3).

6.9 Train Plot

The individual regression models can be plotted for trained standardization pipelines as shown in

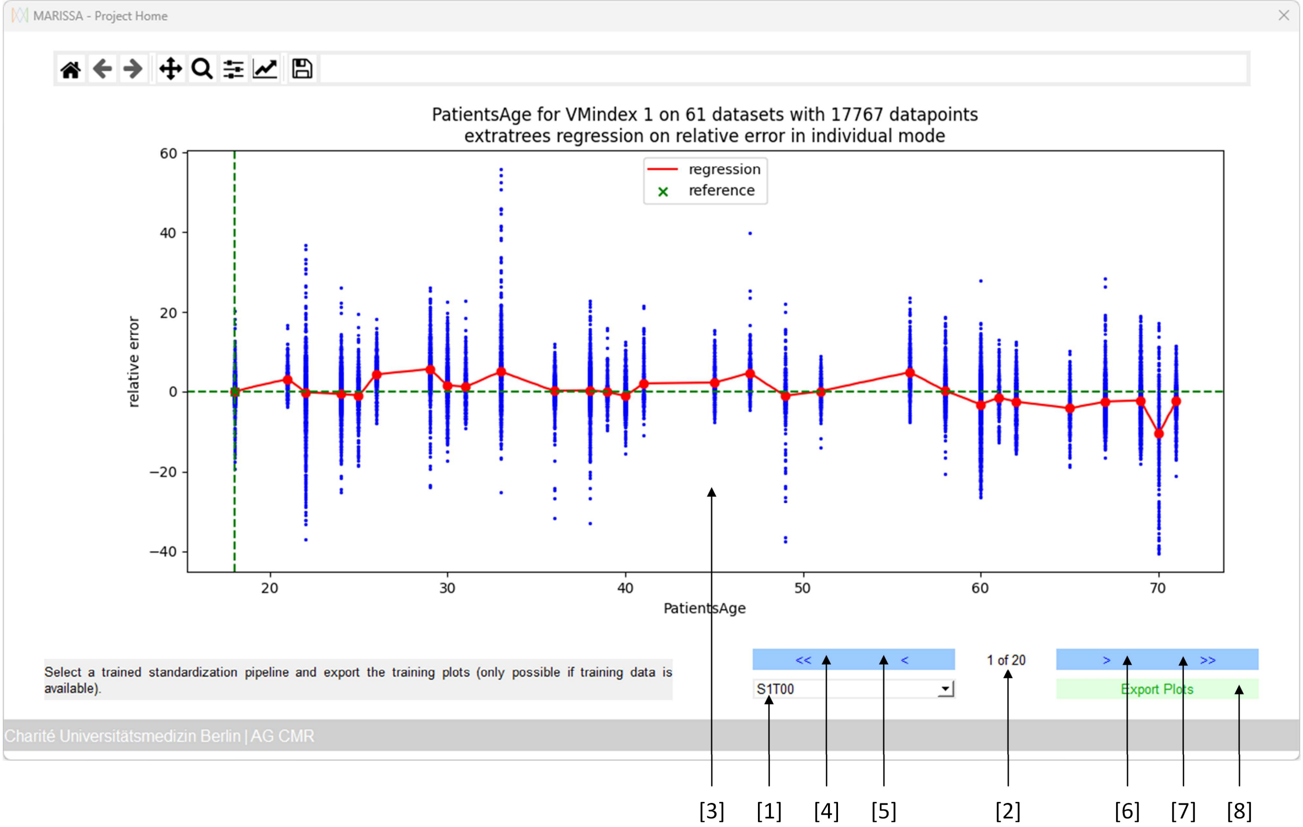

Figure 18: gui_project_plot: [1] standardization pipeline selection, [2] current index of plot with respect to the total number of plots, [3] plot area, [4] go to first, [5] go to previous, [6] go to next, [7] go to last and [8] export plots

The chosen standardization pipeline [1] is used to plot all models [2] in the plot area [3] individually. With the buttons [4] to [7] the user can browse through the plots. Each confounding parameter, each bin and each categorical value has an own plot, therefore the number of plots may exceed the number of confounding parameters. All plots of the selected standardization pipeline [1] are exportable as jpeg files [8].

6.10 Standardize

Trained standardization pipelines are applicable onto external (see section 6.10.1) and internal (6.10.2) data. In any case, the standardization is exported. The export consists for each dataset the original DICOM image, an Excel table and a marissadata file. The Excel file and the marissadata file share basically the same information including the confounding parameter values of the dataset and the segmented T1 values from before until after standardization. The marissadata file is a pickled dictionary, that can be further post-processed in other python tools. Additionally, a progression plot is exported for the mean quantitative value as exemplary shown for three T1 mapping cases in Figure 19.

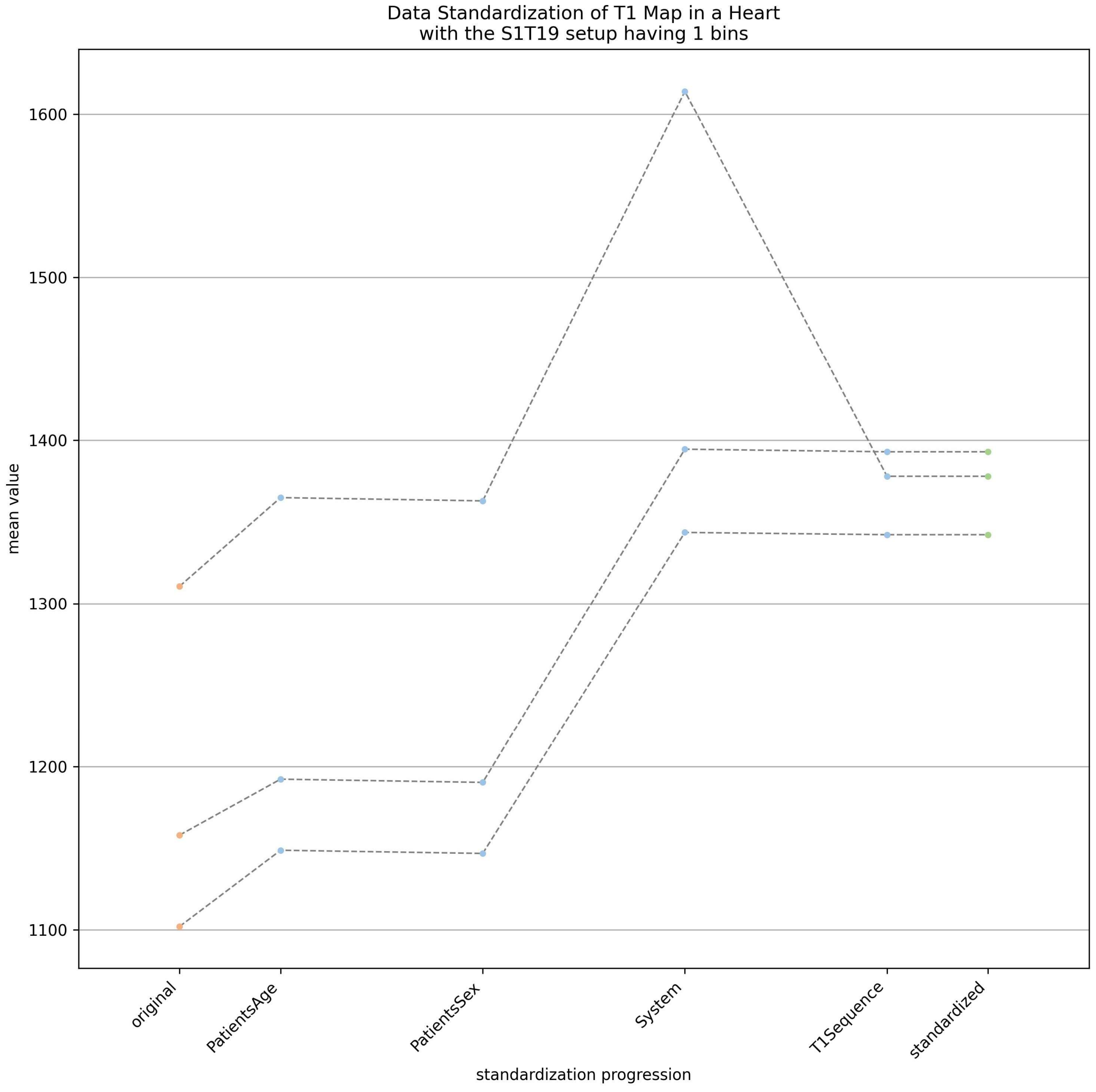

Figure 19: Standardization export: progression plot example

6.10.1 External Data

Figure 20 shows the GUI window to apply standardization on external data.

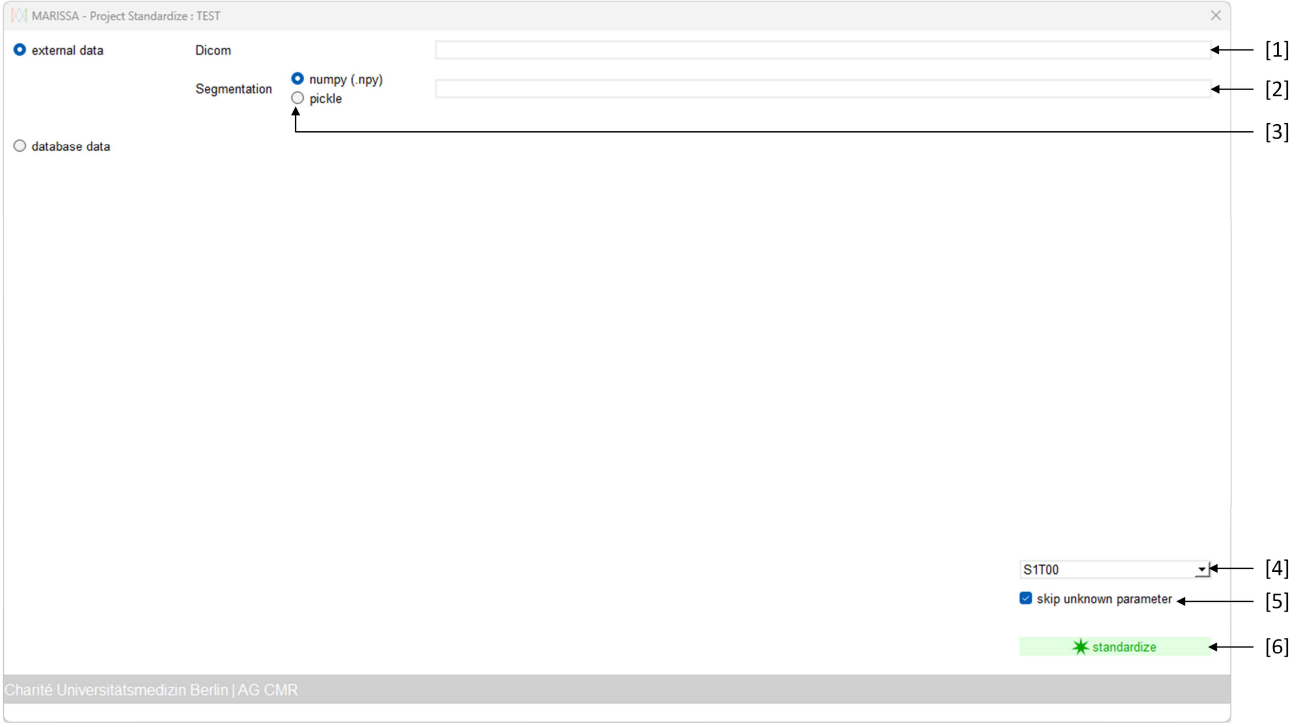

Figure 20: gui_project_standardize – external data: [1] path to DICOM data, [2] path to segmentation data, [3] segmentation data option, [4] standardization pipeline, [5] skipping unknown confounding parameter values (applies to cascaded and individual mode only) and [6] run standardization

The path to external DICOMs must be provided [1]. All files in that directory and subdirectories are checked for being a DICOM file. Furthermore, a path to the segmentation [2] must be provided of either numpy or pickle files [3]. For further information, please refer to section 6.6.2. The filenames of the segmentation must match the SOPinstanceUID of the corresponding DICOM data. Then, the standardization pipeline [4] is chosen and in individual and cascaded mode unknown confounding parameter values can be skipped [5]. This is useful, if for example the specific scanning sequence was not part of the training, but a standardization with respect to the other confounding parameters should be performed. If this box is not checked, then the case is skipped and no standardization is applied. Please note, that only data with matching segmentations are standardized, all others are omitted. The standardization button [6] runs the standardization process and exports the results.

6.10.2 Internal Data

Figure 21 shows the GUI window to apply standardization on internal data.

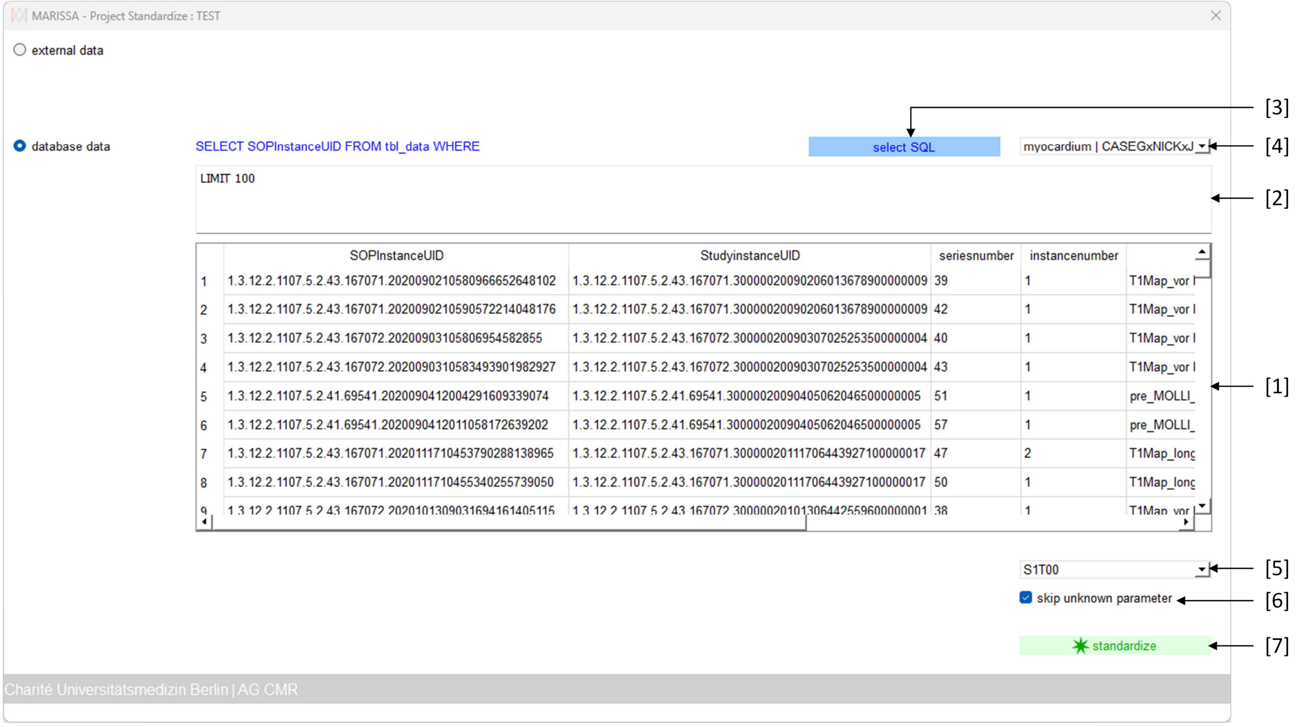

Figure 21: gui_project_standardize – internal data: [1] DICOM data overview, [2] SQL filter, [3] filter button, [4] segmentation selection, [5] standardization pipeline, [6] skipping unknown confounding parameter values (applies to cascaded and individual mode only) and [7] run standardization

The list of all data in the MARISSA project [1] is by default limited to the first 100 occurring cases by the SQL filter [2]. The filter can be adapted and applied by [3]. All data in the list [1] will be used for standardization. The corresponding segmentation needs to be selected [4]. Then, the standardization pipeline [5] is chosen and in individual and cascaded mode unknown confounding parameter values can be skipped [6]. This is useful, if for example the specific scanning sequence was not part of the training, but a standardization with respect to the other confounding parameters should be performed. If this box is not checked, then the case is skipped and no standardization is applied. Please note, that only data with matching segmentations are standardized, all others are omitted. The standardization button [7] runs the standardization process and exports the results.

7 Programming

As MARISSA is a software tool including a GUI, the whole project is structured into subdirectories. Figure 22 shows the directory structure of MARISSA that will be explained in the following in regard of included files and relevant information

Structure				Level
homedirectory				1
	appdata			1.1
		projects		1.1.1
	marissa			1.2
		gui		1.2.1
			designs	1.2.1.1
			images	1.2.1.2
		modules		1.2.2
			clustering	1.2.2.1
			database	1.2.2.2
			regression	1.2.2.3
		toolbox		1.2.3
			creators	1.2.3.1
			tools	1.2.3.2

Figure 22: MARISSA directory structure

7.1 homedirectory (Level 1)

In the homedirectory all relevant data and information of MARISSA is stored. It contains the appdata (see section 7.1.1) and marissa (see section 7.1.2) subdirectories as well as the install.bat, license.lic, MARISSA.bat, README.md and requirements.txt.

7.1.1 appdata (Level 1.1)

The homedirectory\appdata directory contains the MARISSA.sqlite database as well as the projects (see section 7.1.1.1) directory. The MARISSA.sqlite gives information about the current version, contact information and so on. If this file is missing, it will be auto-generated on the next start of the MARISSA software. In order to change the information in the MARISSA.sqlite, the configuration.py (see section 7.1.2.1) must be adapted for the _ConfigDB class.

7.1.1.1 projects (Level 1.1.1)

The homedirectory\appdata\projects is a container directory to store all projects (marissadb files). This directory is read when using MARISSA to list all projects. A deletion of this directory will delete all projects. The import of projects (see section 6.1) will copy them into this directory and vice versa.

7.1.2 marissa (Level 1.2)

The homedirectory\marissa contains all Python files that is necessary to run MARISSA. Running the __init__.py file will start the MARISSA GUI. The files are sorted into the subdirectories: gui (see section 7.1.2.1) for all GUI relevant files, modules (see section 7.1.2.2) for the core implementations of MARISSA and toolbox (see section 7.1.2.3) for all generic functions. Additionally, the scripts subdirectory contains the scripts that were used for the publication of post-hoc standardization of parametric T1 mapping. As this is not necessary for MARISSA, it is not part of this User Manual.

7.1.2.1 gui (Level 1.2.1)

The homedirectory\marissa\gui directory contains all python files of the GUI including the interactions. The respective GUI python files load the GUI design from the designs subdirectory (see section 7.1.2.1.1) with the same filename but the .ui instead of .py extension. The basic.py is a non-used file and represents just the basic Python file structure for the GUIs. This can be used when adding new GUIs. The configuration.py file is important as it contains the runtime information of MARISSA when switching between the GUIs by handing over the configuration Setup object. Additionally the _ConfigDB class is the connection to the MARISSA.sqlite (see section 7.1.1)

7.1.2.1.1 designs (Level 1.2.1.1)

The homedirectory\marissa\gui\designs contains all .ui files representing the GUI windows that were created with the PyQt5 Designer. In order to add or edit GUIs, the PyQt5 Designer is necessary to open these files. All GUI Python files load their respective .ui files. Therefore, the name of controls must match the name of these controls in the .ui file.

7.1.2.1.2 images (Level 1.2.1.2)

The homedirectory\marissa\gui\images is a directory that contains images and logos that are used for the controls buttons and labels. If a control starts with btn_icon_ (for buttons) or lbl_icon_ (for labels), then it adds the image to the control if existing. For example btn_icon_new_dark will add the homedirectory\marissa\gui\images\new_dark.png to the control. This connection is done in the creator_gui.py (see section 7.1.2.3.1)

7.1.2.2 modules (Level 1.2.2)

The homedirectory\marissa\modules has the three subdirectories clustering (see section 7.1.2.2.1), database (see section 7.1.2.2.2) and regression (see section 7.1.2.2.3), which contains the main implementation of MARISSA

7.1.2.2.1 clustering (Level 1.2.2.1)

In the homedirectory\marissa\modules\clustering all available clustering algorithms are implemented. The __init__.py file contains the basic class and functions that are necessary to add a clustering to MARISSA. As the GUI currently do not offer any hyperparameter adaptions of the clustering algorithm apart from the number of clusters, every hyperparameter setting must be implemented as an own file with an own name. Please notice, that the training of a

standardization pipeline copies all necessary information to re-run the training, however, changing the clustering algorithm source code will affect already trained standardization pipelines, so changes should be done with caution.

7.1.2.2.2 database (Level 1.2.2.2)

The homedirectory\marissa\modules\database contains the marissadb.py file, which is the core of the MARISSA. The Module class creates the marissadb file (see section 7.1.1.1) if not existing otherwise it is the connection to it. Figure 23 shows the database structer of the marissadb file.

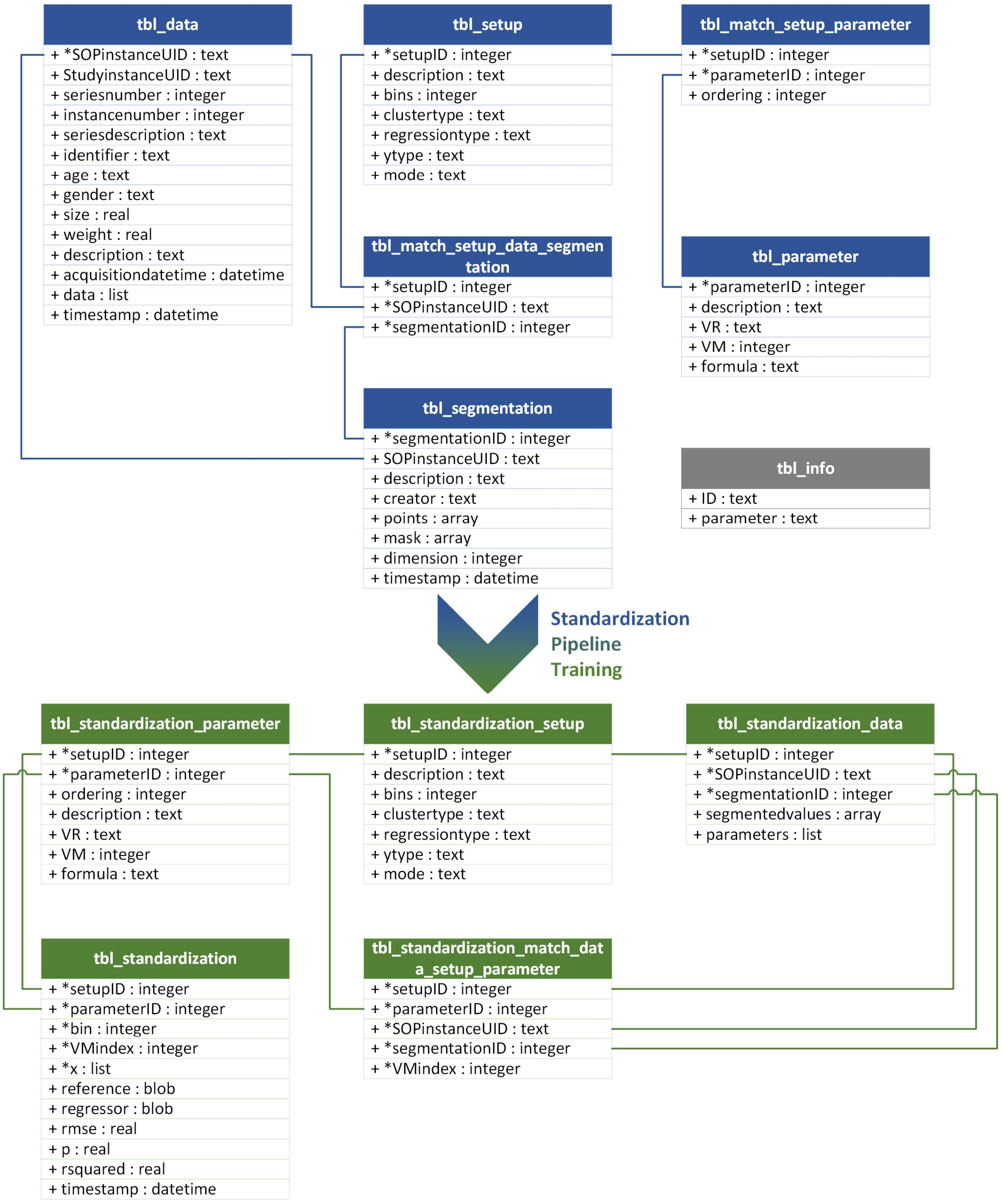

*Figure 23: MARISSA database structure: blue denotes the active part, which is filled and edited by the user, green denotes the trained standardization pipeline. These are filled automatically during training of the standardization pipeline and are separated for consistency. The tbl_info is an overhead information table with no relevance to the functionality itself. The * denotes the primary key fields*

The tables in MARISSA are relational connected, but has two separated parts. The tbl_data, tbl_segmentation, tbl_setup, tbl_parameter, tbl_match_setup_parameter and tbl_match_setup_data_segmentation are filled and edited by user interations through the GUI (see section 5.3). The tbl_standardization_setup, tbl_standardization_data, tbl_standardization_parameter, tbl_standardization_match_data_setup_parameter and tbl_standardization are automatically filled, when the user starts the training of a segmentation

pipeline. All functions, except of simple SQL queries, are implemented in this marissadb.py file.

All tables consist of one or multiple primary key entries. The SOPinstanceUID is read from the DICOM data and represents by definition a unique identifier of the data. The setupID, segmentationID and parameterID are calculated by creating a hash value. The setupID and parameterID has the description only ase base for the hash calculation, while the segmentationID is the hashed value of description, creator and SOPinstanceUID. In any case the upper case of the hash base is used. Consequently, ExampleDeScription and EXAMPLEDESCRIPTION would result in the same hash value. As those are primary keys and therefore unique identifiers, each setup, parameter and segmentation must be unique.

The main functionalities to interact with the tabled of the database are implemented in the marissadb.py as shown in

Table 1: Overview functions to interact with the tables in the SQLite database of a MARISSA project. The x denotes the functions that are implemented in the marissadb.py while (x) shows those that are imported via the import function of tbl_standardization.

**table** **function**	import	insert	update	delete	get
tbl_data	**x**	**x**	**x**	**x**	**x**
tbl_segmentation	**x**	**x**	**x**	**x**	
tbl_parameter		**x**	**x**	**x**	
tbl_setup		**x**	**x**	**x**	
tbl_standardization	**x**	**x**		**x**	
tbl_standardization_setup	**(x)**	**x**			
tbl_standardization_parameter	**(x)**	**x**			
tbl_standardization_data	**(x)**	**x**			
tbl_standardization_match_data_setup_parameter	**(x)**	**x**			

Among additional functions that are implemented in marissadb.py, the following are of interest:

The get_data_parameters returns the parameter values for a given dataset that is referred by the SOPinstanceUID.

The get_data_segmentation returns the segmented values for a given SOPinstanceUI and segmentationID.

The get_standardization calls the get_standardized_values and applies the standardization on a given dataset.

7.1.2.2.3 regression (Level 1.2.2.3)

In the homedirectory\marissa\modules\regression all available regression models are implemented. The __init__.py file contains the basic class and functions that are necessary to add a regression to MARISSA. As the GUI currently do not offer any hyperparameter adaptions of the regression model, every hyperparameter setting must be implemented as an own file with an own name. Please notice, that the training of a standardization pipeline copies all necessary information to re-run the training, however, changing the regression model

algorithm source code will affect already trained standardization pipelines, so changes should be done with caution.

7.1.2.3 toolbox (Level 1.2.3)

The homedirectory\marissa\toolbox includes all generic functions. These are separated in creators (see section 7.1.2.3.1) and tools (see section 7.1.2.3.2). The creators are basic classes from which specific objects inherit while the tools contain single functions.

7.1.2.3.1 creators (Level 1.2.3.1)

The homedirectory\marissa\toolbox\creators contains the basic classes from which the specific objects inherits. Therefore, the class name is always Inheritance.

The creator_configuration.py is the configuration class base (see section 7.1.2.1) and contains the basic functions for save, load, set and reset. The save and load functionality were used in a pre-version but is not necessary anymore as the configuration is newly created on each starting up of MARISSA.

The creator_dialog.py and creator_gui.py are the basic classes for the GUI objects (see section 7.1.2.1). These contain the functions to select a file or directory ss well as show a dialog to the user. Furthermore, window settings are performed, i.e. all text are set to Arial with 10pt. size, as otherwise the font will be too small on UHD displays. Additionally, the icons in the labels and buttons are set (see section 7.1.2.1.2) and widgets starting with mpl_ in the name become replaced by a matplotlib widget as this type of widget is not available in the PyQt5 Designer and needs therefore some self-implementation.

The creator_marissadata.py is the dummy object of the standardization output. The save function will create a pickled dict object file containing all information, such that is directly usable for further processing in other Python tools. Although a load function is implemented, it is currently not used in MARISSA.

The creator_sqlitedb.py is the object connection between MARISSA and the actual sqlite file and contains the necessary functions of connect and disconnect as well as the select and execute functions to run SQL commands. Furthermore, it contains the conversion to compressed byte streams of lists and arrays in order to extend the datatypes in sqlite.

7.1.2.3.2 tools (Level 1.2.3.2)

The homedirectory\marissa\toolbox\tools contains the Python files with general functions.

The tool_excel.py has a Setup and a Reader class to write and read Excel files respectively. A row variable acts as a pointer for the current row, such that writing a new line will write not overwrite prior written lines.

The tool_general.py is a mixed conglomeration of functions that were used in MARISSA but also other independent projects.

The tool_hadler.py are provided functionalities of the Co-Author Thomas Hadler that were necessary, especially to convert between shapely polygons, mask and contours.

The tool_plot.py contains different functions to plot segmentations onto images in matplotlib.

The tool_pydicom.py has some function extensions to work with DICOM data. Some functions are depreciated and not used anymore.

The tool_R.py is the connection between Python and R to either use the NbClust package for optimal cluster size estimation or the FAMD analysis to estimate the impact of individual parameters on a measurement.

The tool_statistics.py provides the calculation of the confusion matrix, the ROC curve analysis and confidence intervals.
